# Modulating endotoxin activity by combinatorial bioengineering of meningococcal lipopolysaccharide

**DOI:** 10.1038/srep36575

**Published:** 2016-11-14

**Authors:** Afshin Zariri, Elder Pupo, Elly van Riet, Jos P. M. van Putten, Peter van der Ley

**Affiliations:** 1 Institute for Translational Vaccinology (Intravacc), Antonie van Leeuwenhoeklaan 9, 3720 AL Bilthoven, the Netherlands; 2 Department of Infectious Diseases and Immunology, Utrecht University, 3584 CL, Utrecht, the Netherlands

## Abstract

*Neisseria meningitidis* contains a very potent hexa-acylated LPS that is too toxic for therapeutic applications. We used systematic molecular bioengineering of meningococcal LPS through deletion of biosynthetic enzymes in combination with induction of LPS modifying enzymes to yield a variety of novel LPS mutants with changes in both lipid A acylation and phosphorylation. Mass spectrometry was used for detailed compositional determination of the LPS molecular species, and stimulation of immune cells was done to correlate this with endotoxic activity. Removal of phosphethanolamine in lipid A by deletion of *lptA* slightly reduces activity of hexa-acylated LPS, but this reduction is even more evident in penta-acylated LPS. Surprisingly, expression of PagL deacylase in a penta-acylated *lpxL1* mutant increased LPS activity, contradicting the general rule that tetra-acylated LPS is less active than penta-acylated LPS. Further modification included expression of *lpxP*, an enzyme known to add a secondary 9-hexadecenoic acid to the 2’ acyl chain. The LpxP enzyme is temperature-sensitive, enabling control over the ratio of expressed modified hexa- and penta-acylated LPS by simply changing the growth temperature. These LPS derivatives display a broad range of TLR4 activity and differential cytokine induction, which can be exploited for use as vaccine adjuvant or other TLR4-based therapeutics.

Lipopolysaccharides (LPS), also known as bacterial endotoxin, are an abundant component of the outer membrane of Gram-negative bacteria. During infection with Gram-negative bacteria, LPS or more precisely the lipid A part of LPS activates the host’s innate immune system^1^,^2^,^3^. This activation occurs through binding of LPS to the pattern recognition receptor Toll-like receptor 4/myeloid differentiation factor 2 (TLR4/MD-2) complex, which starts a signaling cascade leading to cytokine production necessary to clear the infection^3^,^4^. However, overstimulation of this signaling cascade and overproduction of the inflammatory cytokines is detrimental to the host and can lead to life-threatening conditions such as septic shock^5^,^6^.

For complete activation of the TLR4/MD-2 complex, a lipid A structure with six acyl chains and two phosphate groups is critical^7^. However, many bacterial species carry enzymes that can modify their lipid A structure either by changing the number of acyl chains or phosphate groups resulting in altered activation of the TLR4/MD-2 complex^8^, even to the point of being an antagonist instead of an agonist as is observed for the tetra-acylated *E. coli* lipid IVa structure^7^,^9^.

The TLR4/MD-2 complex is unique among the TLR family of receptors because it can signal through both the MyD88 as well as the TRIF pathway. Modified lipid A structures can induce select signaling by preferential recruitment of the MyD88 or TRIF adaptor molecules. Preferential signaling through the TRIF pathway, which triggers production of type I interferons, is thought to be important for vaccine adjuvants^10^,^11^. Monophosphoryl lipid A (MPLA) is an example of a modified lipid A that triggers a TRIF-biased signaling^11^. MPLA is a heterogeneous lipid A mixture from *Salmonella Minnesota*, which has been chemically detoxified and is approved for the use as adjuvant in some vaccines^12^. The main component of MPLA consists of a hexa-acylated 4′-monophosphoryl lipid A. Use of MPLA has the disadvantage that for its production a chemical treatment is needed in addition to LPS isolation from the bacteria and it only consists of the lipid A portion of LPS, making it completely water insoluble, whereas complete LPS can be administered in water.

*Neisseria meningitidis* typically produces hexa-acylated LPS with phosphate and phosphoethanolamine groups appended to the 1 and 4′ position of the lipid A^13^,^14^. Heterologous expression of LPS modifying enzymes such as PagL or deletion of lipid A biosynthesis enzymes such as LpxL1 and LpxL2 has been used to detoxify the highly active meningococcal LPS^13^,^15^. Deletion of LpxL1 was shown to be an advantageous method for detoxifying meningococcal LPS when making meningococcal outer membrane vesicle vaccines, without the need to use a detergent to reduce excess LPS-related reactogenicity^16^. However, the activity of this modified LPS has been reduced to the extent that it barely induces any activation of the TLR4/MD-2 complex on human cells, making it less applicable as a stand-alone vaccine adjuvant^17^. Heterologous expression of *pagL* in *N. meningitidis* results in a different attenuated penta-acylated LPS structure, which is still capable of inducing TLR4 activation and induces a TRIF-biased cytokine production on a human monocytic cell line^13^. The challenge of LPS-based adjuvants is finding the optimal balance between retaining a sufficient amount of immune activation while limiting toxic side effects. In the present study, we have used heterologous expression of LPS modifying enzymes in combination with targeted deletion of lipid A biosynthesis genes to collect a diverse set of meningococcal LPS structures with a broad range of TLR4/MD-2 activation capacities.

## Results

### Bioengineering of modified LPS structures

**Figure 1:**
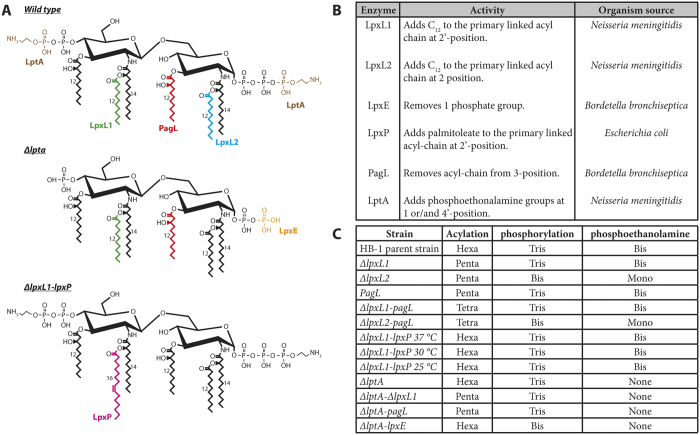
Applied lipid A modification enzymes. The structural changes made by the various combinations of modifying enzymes are depicted in color (**A**). LpxL1 (green), LpxL2 (blue), LpxP (pink) and LptA (brown) all add the corresponding group to the molecule, whereas PagL (red) and LpxE (orange) remove the group. The abbreviation of the enzymes, organism source and activity are presented (**B**) and the acylation pattern and number of phosphate and phosphoethanolamine groups for each strain are indicated (**C**).

LPS mutants in *N. meningitidis* were constructed in the HB-1 derivative of strain H44/76. Mass spectrometric analysis demonstrated that HB-1 expresses a hexa-acylated, tri-phosphate, bis-phosphoethanolamine lipid A structure (see below). To construct a diverse set of LPS mutants in strain HB-1, we inactivated the autologous genes encoding for the LPS enzymes LptA, LpxL1 and LpxL2 and heterologously expressed the LpxE, LpxP and PagL LPS enzymes (Fig. 1A,B). In addition, combinations of deletion of autologous genes and expression of heterologous enzymes were constructed. This approach resulted in 10 LPS mutant strains as depicted in Fig. 1C.

**Figure 2:**
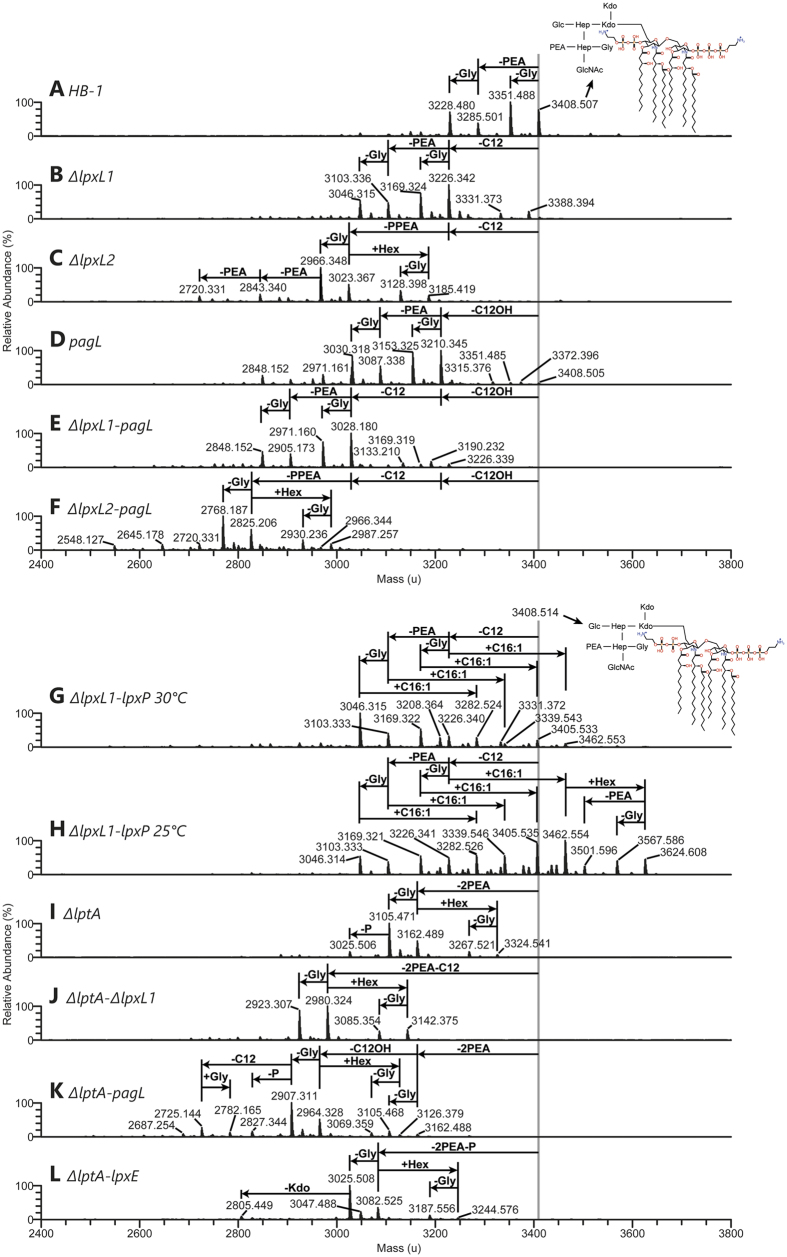
Charge deconvoluted ESI-FT mass spectra of LPS. The charge deconvoluted ESI-FT mass spectra of the LPS isolated from twelve different strains of *Neisseria meningitidis* are shown as follows: parent HB-1 strain (**A**), Δ*lpxL1* (**B**), Δ*lpxL2* (**C**), *pagL* (**D**), Δ*lpxL1*-*pagL* (**E**), Δ*lpxL2*-*pagL* (**F**), Δ*lpxL1*-*lpxP* cultured at 30 °C for 5 h (**G**) or at 25 °C overnight (**H**), Δ*lptA* (**I**), Δ*lptA*-Δ*lpxL1* (**J**), Δ*lptA*-*pagL* (**K**) and Δ*lptA*-*lpxE* (**L**). A simplified representation of the LPS structure assigned to the ion of 3408.507 u is included in mass spectrum (**A**). The vertical line at a mass of 3408.514 u, which corresponds to the calculated molecular mass of this latter LPS species, is used as a reference to indicate LPS composition assigned to other ion signals. See Supplementary Table 1 for detailed LPS composition proposals. All annotations refer to monoisotopic masses of the neutral molecules.

For the expression of LpxE (Protein ID: CAE41138.1) we initially cloned an *lpxE* homologue from *Bordetella pertussis*. However, expression of this gene in HB-1 or its *lptA* mutant derivative did not result in any LPS structural changes as determined by mass spectrometry. As an alternative the *lpxE* (Genbank accession number: WP_003809405.1) homologue from *Bordetella bronchiseptica*, which exists as a pseudogene in *B. pertussis*, was cloned and expressed in a *ΔlptA* mutant strain. This resulted in the loss of a phosphate group in the lipid A and was included in our panel of LPS mutant strains (Fig. 2L).

LpxP (Genbank accession number: U49787.1), an enzyme known to add a secondary 9-hexadecenoic acid (C16:1) to the 2′ acyl chain in *E. coli*^18^, was expressed in the *N. meningitidis ΔlpxL1* mutant strain. The LpxL1 enzyme adds a secondary acyl chain to the same position as LpxP, so we reasoned that its absence could increase the efficiency of any C16:1 addition. This modification was expected to create a hexa-acylated lipid A structure different from the original by carrying the longer, mono-unsaturated C16:1 secondary acyl chain in the 2′ position instead of C12. When LpxP was expressed in the *ΔlpxL1* mutant strain at 37 °C this resulted in a very faint addition of C16:1. However, C16:1 is added onto *E. coli* LPS only at 12 °C, so for this reason we grew the bacteria at lower temperatures. Cultivation of meningococci below 25 °C is, unlike in *E. coli*, not possible, but at 25 °C and 30 °C we already found a much higher relative abundance of the LpxP hexa-acylated lipid A structure carrying the additional C16:1, with 25 °C resulting in the highest efficiency (at least 50% relative abundance) (Fig. 2H).

### Mass spectrometric characterization of modified LPS

The charge-deconvoluted ESI-FT mass spectra of intact LPS isolated from the constructed *N. meningitidis* mutants are shown in Fig. 2. The mass spectrum of LPS of the HB-1 (*galE*-) parent strain (Fig. 2A), displayed an ion signal of 3408.507 u consistent with LPS comprised of wild-type hexa-acyl lipid A carrying three phosphate (P) and two phosphoethanolamine (PEA) groups and an L3-immunotype oligosaccharide structure substituted with a glycine (Gly) residue and truncated at the proximal galactose (Gal) of its alpha chain due to inactivation of the *galE* gene (Mcalc. = 3408.514 u, see Supplementary Table 1 for LPS composition proposals). Accompanying ion peaks of 3351.488, 3285.501 and 3228.480 u (Fig. 2A) corresponded to LPS species which lack Gly (Δmeas. = −57.019 u), carry one less PEA group in the lipid A (Δmeas. = −123.006 u) or both (Δmeas. = −180.027 u), respectively. Therefore, the chemical heterogeneity of the LPS from HB-1 (*galE*-) strain was caused by variation of lipid A phosphorylation and oligosaccharide non-stoichiometric substitution with glycine. Composition proposals based on mass spectra of intact LPS were additionally supported by FT-MS analysis of LPS fragment ions corresponding to lipid A and oligosaccharide moieties, which were generated by in-source collision induced dissociation (SID) of intact LPS. For instance, SID FT mass spectra of LPS from HB-1 (*galE*-) strain displayed fragment ions of 1916.098 and 2039.106 u corresponding to hexa-acyl lipid A species with 2 and 3 PEA groups (Mcalc. = 1916.100 and 2039.109 u, respectively) and a fragment ion of 1369.404 u corresponding to the dehydrated derivative of the oligosaccharide moiety described above (Mcalc. = 1369.406 u). Fragmentation analyses of LPS derived from other strains of *N. meningitidis* described here showed that different types of LPS carry the same oligosaccharide moieties (PEA_**1**_•Hex_**1**_•Hep_**2**_•HexNAc_**1**_•Kdo_**2**_•Gly_**1**_), with the exception of some LPS species, which lack a glycine or carry a second hexose residue (Hex) (Supplementary Table 2). Consequently, other differences observed between the LPS species, such as in the number of PEA and P groups, were attributed to changes in the composition of the lipid A (Supplementary Table 2).

Analysis of the intact LPS from the *ΔlpxL1* mutant revealed that he main ion peaks of the mass spectrum (3046.315, 3103.336, 3169.324 and 3226.342 u, Fig. 2B) had shifted compared to the 4 main ion signals of the LPS from the parent HB-1 (*galE*-) strain (Fig. 2A) by -182.165 u. This is in agreement with the lack of a dodecanoic acid (C12) (Δcalc. = −182.167 u) in the lipid A after deletion of the *lpxl1* gene.

The mass spectrum of the LPS from the *ΔlpxL2* mutant displayed ion peaks of 3023.367, 2966.348, 3185.419 and 3128.398 u (Fig. 2C), which are consistent with the loss of a C12 fatty acyl chain together with PPEA from the lipid A (Δcalc. = −385.142 u) in combination with non-stoichiometric substitution of the oligosaccharide with Gly (Δcalc. = 57.021 u) or a second hexose (Δcalc. = 162.053 u). This is in agreement with effective deletion of the *lpxL2* gene. It is worthy to note that deletion of the *lpxL2* gene not only led to the loss a C12 fatty acyl chain, as observed earlier upon deletion of the *lpxL1* gene, but also resulted in the loss of a P and a PEA group from the lipid A.

The ion peaks in the mass spectrum of the LPS from the *pagL* mutant (3210.345, 3153.325, 3087.338 and 3030.318 u, Fig. 2D) were found to be shifted by −198.163 u from the 4 main ion peaks of the LPS from the parent HB-1 strain. This is in agreement with efficient removal of a 3-hydroxy-dodecanoic acid (C12OH) (Δcalc. = −198.162 u) from the lipid A by the PagL enzyme. Nonetheless, display of minor ion peaks of 3408.505 and 3351.485 u (Fig. 2D) corresponding to unmodified hexa-acyl LPS species indicated that LPS 3-O-deacylation activity of the PagL enzyme could not fully exhaust the hexa-acyl lipid A substrate.

The 4 main ion signals in the mass spectrum of the LPS from the *ΔlpxL1-pagL* mutant (3028.180, 2971.160, 2905.173 and 2848.152 u, Fig. 2E) differed by −380.328 u from the 4 main ion signals of the LPS from the HB-1 strain. This is accordance with lack of a C12 and a C12OH in the lipid A of the *ΔlpxL1-pagL* mutant (Δcalc. = −380.329 u). The absence of ion signals corresponding to LPS carrying two C12 acyl chains indicates that the deletion of the *lpxL1* gene resulted in complete removal of a single C12 from the lipid A (see Supplementary Table 1 for detailed LPS composition proposals). In contrast, minor ion signals of 3226.339 and 3169.319 u were present in the mass spectrum of the LPS from the *ΔlpxL1-pagL* mutant, which correspond to penta-acyl LPS species carrying two C12OH acyl chains. This indicates that a low level of LPS molecules was not 3-O-deacylated by the PagL enzyme.

The mass spectrum of the LPS from the *ΔlpxL2-pagL* mutant showed an ion peak of 2825.206 u (Fig. 2F) that was shifted by −583.301 u from the ion signal of 3408.507 u of the mass spectrum of the LPS from the parent HB-1 strain (Fig. 2A). This fits the expected loss of a C12OH, a C12 and PPEA from the lipid A (Δcalc. = −583.304 u). Other ion signals of 2768.187, 2930.236 and 2987.257 u (Fig. 2F) are consistent with non-stoichiometric substitution of the oligosaccharide with Gly or a second Hex.

Comparison of the mass spectrum of the LPS from the *ΔlpxL1-lpxP* mutant grown at 30 °C (Fig. 2G) with that of the LPS from the *ΔlpxL1* mutant (Fig. 2B) revealed that the LPS from the *ΔlpxL1-lpxP* mutant contained not only the main LPS species that were present in the LPS from the *ΔlpxL1* mutant (3046.315, 3103.333, 3169.322 and 3226.340 u, Fig. 2G), corresponding to penta-acyl LPS lacking a C12, but also LPS species (3282.524, 3339.543, 3405.533 and 3462.553 u, Fig. 2G) that shifted in the spectrum to higher mass values by 236.211 u. This is in agreement with incorporation of a 9-hexadecenoic acid (C16:1) to the lipid A. Therefore, this preparation comprised a mixture of penta-acyl LPS that lacks a C12 and hexa-acyl LPS that lacks a C12 and additionally carry a C16:1.

The mass spectrum of the LPS from the *ΔlpxL1-lpxP* mutant cultured at 25 °C (Fig. 2H) showed ion signals corresponding to hexa-acyl LPS lacking a C12 and carrying additionally a C16:1 (3282.526, 3339.546, 3405.535 and 3462.554 u, Fig. 2H), which were of a higher relative abundance as compared to the same signals in the spectrum of the LPS from the *ΔlpxL1-lpxP* mutant grown at 30 °C. Furthermore, other ion peaks corresponding to hexa-acyl LPS carrying a C16:1 were displayed which arose from elongation of the oligosaccharide with a second Hex (3624.608 u) or the latter in combination with the loss of Gly substitution (3567.586 u) and the loss of a PEA group from the lipid A (3501.596 u) (Fig. 2H).

The ion peak of 3162.489 u in the mass spectrum of the LPS from the *ΔlptA* mutant (Fig. 2I) differed by −246.018 u from the ion signal of 3408.507 u of the mass spectrum of the LPS from the parent HB-1 strain (Fig. 2A). This points to the loss of two PEA groups from the lipid A (Δcalc. = −246.017 u). Other ion signals corresponded to LPS species that in addition to lacking PEA in the lipid A either lacked Gly in the oligosaccharide (3105.471), contained a second Hex in the oligosaccharide (3324.541) or contained a second Hex and lacked Gly in the oligosaccharide (3267.521 u) (Fig. 2I).

The mass spectrum of the LPS from the *ΔlptA-ΔlpxL1* mutant displayed ion peaks of 2980.324, 2923.307, 3142.375 and 3085.354 u indicating the loss of 2PEA and a C12 from the lipid A (Δcalc. = −428.184 u) combined with non-stoichiometric substitution of the oligosaccharide with Gly or a second Hex (Fig. 2J).

The main ion signals of the mass spectrum of the LPS from the *ΔlptA-pagL* mutant (2964.328 and 2907.311 u, Fig. 2K) are consistent with the loss of 2PEA and a C12OH from the lipid A (Δcalc. = −444.179 u) together with non-stoichiometric substitution of the oligosaccharide with Gly (Δcalc. = 57.021 u). Minor ion peaks of 3105.468 and 3162.488 u were observed corresponding to hexa-acyl LPS species which lost only 2PEA from the lipid A, indicating a low level of incomplete LPS 3-O-deacylation by the PagL enzyme.

Finally, the mass spectrum of the LPS from the *ΔlptA-lpxE* mutant showed 2 main ion peaks of 3082.525 and 3025.508 u consistent with loss of 2PEA and P from the lipid A (Δcalc. = −325.983 u) in combination with non-stoichiometric substitution of the oligosaccharide with Gly (Fig. 2L). In addition, MS/MS spectra of the main lipid A fragment ion produced by in-source collision-induced dissociation of LPS were consistent with the presence of a P group at both the 1 and 4′ positions of the lipid A (data not shown). Therefore, the activity of the LpxE enzyme consisted in removal of one of the three P groups present in lipid A producing bisphosphorylated lipid A species with a P group on each side of the diglucosamine backbone.

### TLR4 stimulation by the LPS mutant strains

**Figure 3:**
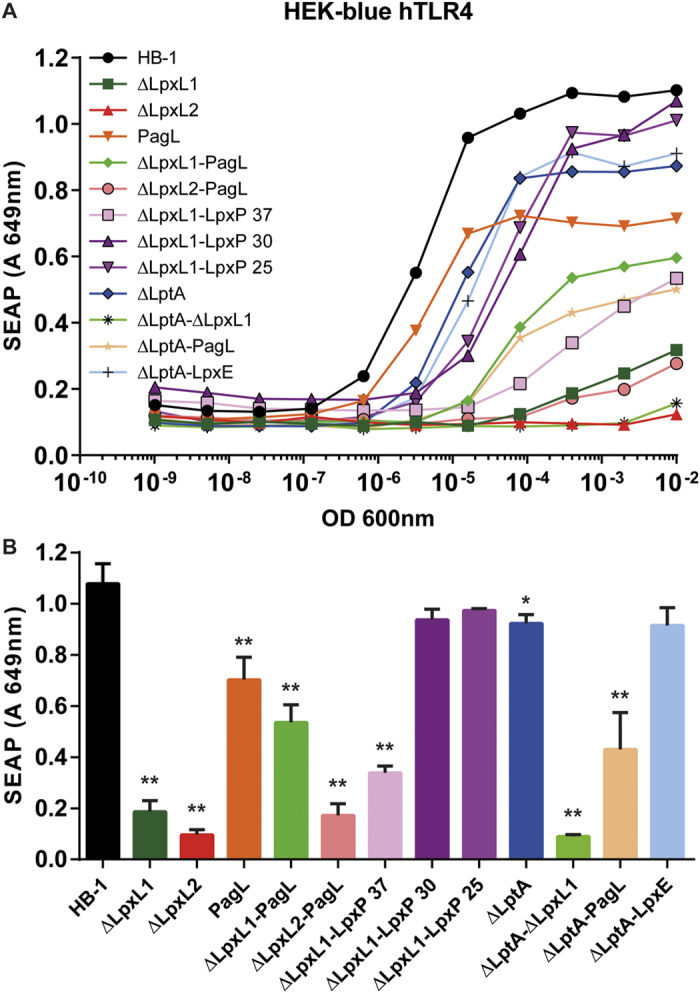
TLR4 activation by *N. meningitidis* strains. HEK-blue hTLR4 cells were stimulated with 5-fold serial dilutions of heat-inactivated *N. meningitidis* for 20 h. TLR4 activation was measured by detection of secreted alkaline phosphatase. Results of serial dilutions are depicted in a line graph (**A**) and for a single OD_600 nm_ of 0.0004 in a bar graph (**B**). Data are expressed as mean values or mean ± SD of three independent experiments. Statistical significance was determined with an ANOVA test comparing against HB-1. *p < 0.05.; **p < 0.001.

To determine the scope of TLR4 activation by the entire set of lipid A mutant structures, an initial screening was done using HEK-Blue human TLR4 cells. These cells express human TLR4, MD-2, and CD14 and contain a nuclear factor kappa-light-chain-enhancer of activated B cells (NF-κB) and activator protein 1 (AP-1) dependent secreted embryonic alkaline phosphatase (SEAP) reporter gene. Stimulation of cells with serial dilutions of the different LPS mutants yielded a wide range of TLR4 activities (Fig. 3), with HB-1 inducing strongest TLR4 activation and *ΔLpxL2* bacteria yielding lowest levels of activation. The other LPS mutants showed intermediate TLR4 stimulating activity (Fig. 3). A particularly notable result was that the absence of phosphoethanolamine in the *ΔlptA* strain resulted in reduced TLR4 activation in both the hexa-acylated wild type strain and the penta-acylated *ΔlpxL1* and *pagL* backgrounds. Induction of LpxE in the *ΔlptA* strain showed similar TLR4 activation as *ΔlptA* strain, which was slightly less than the HB-1 wild type strain. The expression of LpxE in the *ΔlptA* strain did not show major additional effects compared to the *ΔlptA* mutant strain thus indicating that the reduction of three phosphates to two in the lipid A structure did not have a major effect on TLR4 signaling.

Expression of LpxP at 25 °C in combination with deletion of LpxL1 resulted in a heterogeneous hexa- and penta-acylated structure-LPS expressing strain with a slightly reduced TLR4 activating potential compared to the wild type bacteria. Cultivation of this strain at 30 °C resulted in less hexa-acylated lipid A and even slightly less TLR4 activity.

Surprisingly, when the *ΔlpxL1* strain was combined with expression of PagL, reducing the penta-acylated lipid A structure to a tetra-acylated form, an increase of TLR4 activity was obtained. This was unexpected as tetra-acylated lipid A structures typically act as a TLR4 antagonists as reported for *E. coli* lipid IVa^7^,^9^,^19^. Interestingly, PagL-mediated deacylation of wildtype LPS instead reduced its activity.

### Human TLR4 stimulation using purified mutant LPS

**Figure 4:**
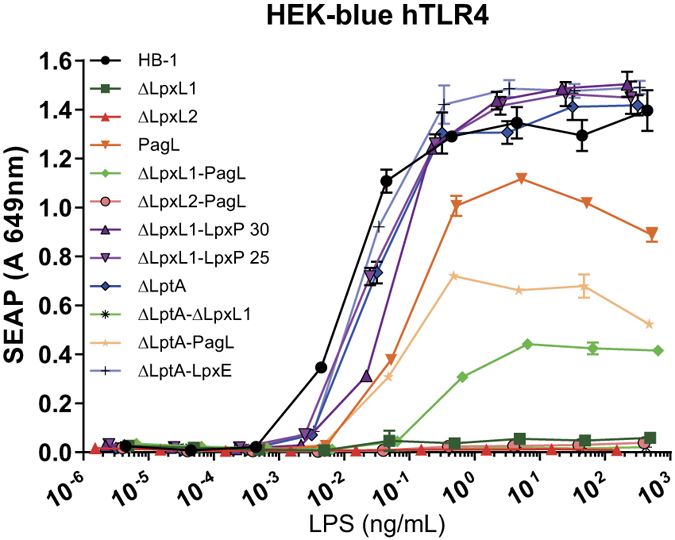
TLR4 activation by purified LPS. HEK-blue hTLR4 cells were stimulated with 10-fold serial dilutions of 12 different LPS mutants. TLR4 activation was measured by detection of secreted alkaline phosphatase. Data shown are from one representative experiment out of three independent experiments with similar outcomes and are depicted as the mean ± SEM of triplicates.

We also purified LPS from all strains and used the molecules to stimulate HEK-Blue TLR4 cells to confirm our initial findings with whole bacteria. Purified LPS generally yielded similar results as those obtained with intact bacteria although purified LPS from *ΔlpxL1, ΔlptA-ΔlpxL1, ΔlpxL2* and *ΔlpxL2-pagL* mutants showed almost no induction of TLR4 activity and were barely distinguishable from each other (Fig. 4), whereas the corresponding bacteria displayed low but distinct TLR4 activities above the background. In addition, a higher concentration of purified penta-acylated *pagL* LPS was needed for activation of TLR4 than with all the hexa-acylated LPS derivatives, but with whole bacteria stimulation, a lower optical density was necessary for the *pagL* strain to induce TLR4 activity than the other hexa-acylated mutant strains (Figs 3 and 4). However, the maximum amount of alkaline phosphatase secretion was still lower for the *pagL* mutant strain compared to the hexa-acylated mutant strains. Of note, the three LPS mutants *ΔlpxL1-pagL, pagL* and *ΔlptA-pagL* had substantially reduced activating capabilities when compared to the wild type LPS, but still induced activation above the background level of unstimulated cells (Fig. 4).

### Cytokine induction by the purified mutant LPS

**Figure 5:**
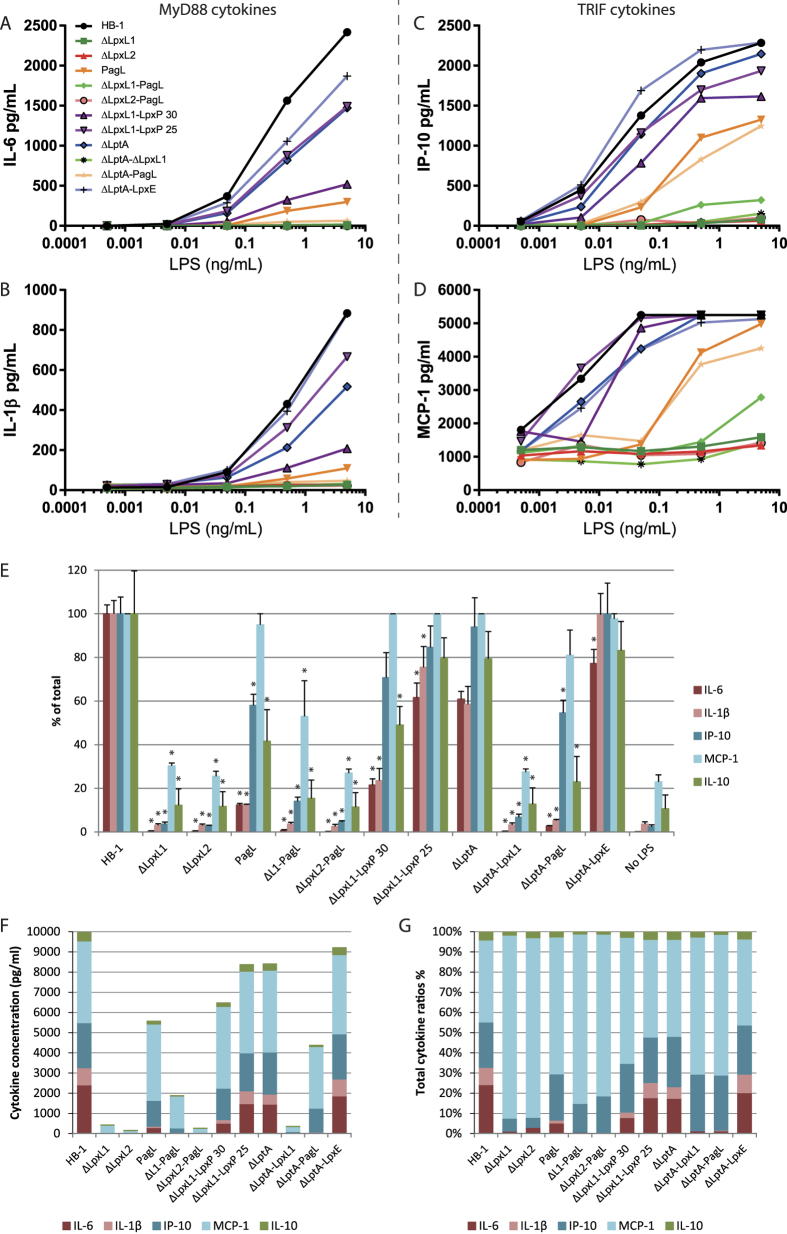
Cytokine release of MM6 cells stimulated with purified LPS. MM6 cells were incubated with 10-fold serial dilution of different LPS mutants for 20 h. IL-6 (**A**), IL-1β (**B**), IP-10 (**C**), MCP-1 (**D**) production was measured by ELISA. IL-6 and IL-1β are considered MyD88 dependent cytokines and IP-10 and MCP-1 are more TRIF dependent. Cytokine levels of MM6 cells stimulated with 5 ng/ml LPS are also presented as percentages of the HB-1 strain (**E**) and cytokine ratios in concentration (**F**) and percentages (**G**). For the cytokine ratios (**F**,**G**) the background without LPS stimulation was subtracted. Data shown are depicted as the mean values of two independent experiments. Statistical significance was determined with a 2-way ANOVA test comparing against HB-1. *p < 0.05.

The cytokine induction profile of the modified LPS structures was investigated in the human monocytic cell line Mono Mac 6 (MM6). The concentration of secreted MyD88 dependent cytokines IL-6 (Fig. 5A) and IL-1β (Fig. 5B) and TRIF dependent cytokines interferon gamma-induced protein 10 (IP-10) (Fig. 5C) and monocyte chemotactic protein-1 (MCP-1) (Fig. 5D) were determined after 20 h of stimulation with purified LPS (Fig. 5). The possible contribution of minor protein contamination in LPS samples to the observed responses was excluded as activation of a HEK-hTLR2 cell line by the LPS samples was negligible in the range of LPS concentrations tested (data not shown).

A wide variety of cytokine levels was determined from the different LPS structures, with the highest levels being produced by the HB-1 wild type hexa-acylated LPS and all other LPS ranging from close to wild type until virtually zero cytokine induction as seen for *ΔlpxL2* LPS. Besides quantitative differences in cytokine induction, we also observed qualitative differences with LPS structures causing reduced levels of certain cytokines, but still capable of producing others. Some examples are *pagL* and *ΔlptA-pagL* LPS, which displayed a reduced capacity to induce the production of MyD88 dependent pro-inflammatory cytokines IL-6 and IL-1β (10% or less of the levels induced by wild-type LPS, respectively), but retained most of the ability to induce the secretion of TRIF dependent IP-10 (~50%) and MCP-1 (~90%). Interestingly, differences were observed between *ΔlpxL1*-*lpxP* grown at 30 °C and 25 °C, with *ΔlpxL1*-*lpxP* grown at 30 °C producing 20–30% IL-6 and IL-1β and 60–80% of those cytokines at 25 °C, whereas IP-10 and MCP-1 induction were similar. These results emphasize how LPS bioengineering can provide a wide range of agonists to fine-tune cytokine release.

## Discussion

Although LPS has great potential as an adjuvant, adverse effects keep being a concern. Finding the optimal balance between adjuvant activity and minimal toxic effects requires the development of new LPS derivatives. Here we report a collection of novel meningococcal LPS structures inducing a broad range of TLR4 responses and differential cytokine patterns. These combinatorial bioengineered LPS mutants can be used as part of a whole cell vaccine, OMV vaccine or as purified LPS or lipid A molecule. OMVs of *N. meningitidis* are being actively investigated as potential vaccines and have been already approved for use in humans as a component of the Bexsero vaccine against serogroup B meningococcal disease^20^,^21^. Attenuated *ΔlpxL1* LPS is under investigation as constituent of meningococcal OMV vaccines and combinatorial bioengineering of LPS is a safe method to detoxify OMVs^16^,^22^. In addition, in an immunization study in mice purified *ΔlpxL1* LPS retained similar adjuvant activity compared to wild type meningococcal LPS, but with reduced toxicity^15^.

The modified LPS molecules *ΔlpxL1, ΔlpxL2* and *pagL* all result in a reduced TLR4 activity compared to the parent strain^13^,^15^. This was expected because of the reduction of the number of acyl chains in the LPS from hexa to penta. Surprisingly, the expectation that tetra-acylated LPS is always less active than penta-acylated LPS is challenged by our results. Tetra-acylated lipid IVa of *E. coli* is a known antagonist of the human TLR4/MD-2 complex^7^,^9^,^19^. Yet, we show that meningococcal tetra-acylated *ΔlpxL1-pagL* LPS is more active than the penta-acylated *ΔlpxL1* LPS, whereas tetra-acylated *ΔlpxL1-ΔlpxL2* LPS did not yield detectable activity (data not shown). Stimulation with *ΔlpxL2-pagL* whole bacteria that also carry a tetra-acylated LPS again increased TLR4/MD-2 activity compared to its penta-acylated *ΔlpxL2* parent strain, although purified LPS from both the *ΔlpxL2-pagL* and *ΔlpxL2* were inactive. Together these findings indicate that removal of C12OH from the 3-position by PagL in combination with deletion of a secondary acyl chain resulting in tetra-acylated lipid A yields a higher TLR4 activity compared to sole removal of the secondary acyl chain or both secondary acyl chains. By contrast, PagL deacylation in wildtype LPS results in a decrease of TLR4 activity. LPS structures engineered in *E. coli* by Needham *et al*.^23^ included a tetra-acylated LPS consistently being more active than its penta-acylated parent strain. Although this structure had a different acyl chain distribution and chain length than our tetra-acylated structure *ΔlpxL1-pagL*, through expression of PagL and deletion of LpxM it also resulted in removal of C12OH from the 3-position and deletion of a secondary acyl chain, respectively.

Interestingly, introduction of LpxP from *E. coli* into the *N. meningitidis lpxL1* strain conferred a temperature-sensitive lipid A modification to *N. meningitidis*, with a secondary C12 being replaced by C16:1. Since conservation of temperature-sensitive gene expression signals is unlikely, this means that the enzyme itself is most active at lower temperatures. Selection of a temperature of 25 or 30 °C for culture of the *ΔlpxL1-lpxP* strain influenced the amount of hexa-acylated LPS species present in the mixture of penta- and hexa-acylated LPS produced by this mutant, with the lower temperature leading to the highest degree of substitution. The temperature sensitivity of the LpxP enzyme thus enables the preparation of penta- and hexa-acylated LPS mixtures in a controlled manner. By selecting the time and/or temperature that the mutant strain is grown, it is feasible to increase or decrease the amount of hexa-acylated lipid A structure and thereby the TLR4 activity and cytokine profile. This provides a new approach of fine-tuning the immunological properties of meningococcal OMV vaccines.

In addition, we have obtained new insight in the specificity of the LpxE enzyme. Previously, the *lpxE* gene from *Francisella tularensis* or *Francisella novicida* expressed in *E. coli* was shown to be specific for the removal of the P group in the 1-position^23^,^24^. We have found that the *lpxE* homologue from *B. bronchiseptica* removed only one P group from the total of three present in the lipid A of *N. meningitidis*. MS/MS spectra of the lipid A from *ΔlptA-lpxE* mutants were consistent with the presence of a P group at both the 1 and 4′ positions of the lipid A. In addition, removal of the P group was only seen in double *ΔlptA-lpxE* mutants, therefore only in the absence of PEA substitution of the lipid A. Thus, it is likely that the presence of PEA prevents LpxE from removing the P group. Most likely, the newly described LpxE enzyme is a pyrophosphatase, only catalyzing hydrolysis between two phosphate groups. The absence of PEA in the lipid A through deletion of the *lptA* gene resulted in a reduced TLR4/MD-2 activity. This concurs with earlier observations by John *et al*.^25^ that show a significant reduction of TNFα release by THP-1 cells upon stimulation with LptA lacking strains. Here we showed that reduction of the activity is even more apparent when stimulated with penta-acylated *ΔlptA-pagL* LPS or whole bacteria.

Interestingly, our results indicate that the absence of a C12 fatty acyl chain by deletion of LpxL2 is accompanied by loss of a single P group and PEA group. Possibly, loss of this secondary acyl chain may interfere with efficient P or PEA addition. This was not observed in previous structural analysis of the *lpxL2* mutant, due to isolation of lipid A by an acid hydrolysis method before mass spectrometric analysis, which can result in the loss of P groups from the lipid A^15^. In the present study, we used complete LPS molecules without introducing any deleterious chemical modifications for mass spectrometric analysis, giving us the possibility to observe new phosphorylation changes of the lipid A.

Several of the constructed attenuated LPS structures did not only need a higher concentration to induce TLR4 stimulation, but also did not reach the maximum level of activation observed for the parent strain. This was most apparent for *pagL* LPS. The reason for this phenomenon is unclear, but could be due to instable dimerization of the LPS-TLR4-MD2 receptor complex at the cell surface but stable dimerization inside the cell, and/or to a less stable dimerization with high concentrations of the particular LPS. In addition, certain LPS species showed no activation at all and could potentially have antagonistic features, and might therefore serve as a TLR4 blocking drug. Indeed, meningococcal *ΔlpxL1, ΔlpxL2* and *pagL* penta-acylated LPS can block the TLR4 response when administered together with hexa-acylated wild type meningococcal or *E. coli* LPS^13^,^26^.

In the present study, we have used combinatorial bioengineering in meningococci to produce a range of LPS species with a broad array of TLR4 activity and cytokine profile. Together, they cover the whole range of activity from wildtype maximum to practically zero (Fig. 4). The application of these structures can be very broad, from inclusion into vaccines as adjuvants to their use in various forms of immunotherapy. OMVs from the mutant strains can be isolated, using the modified LPS as an improved internal adjuvant. In addition, they can enable innovative forms of immunotherapy which have been described or suggested for LPS, such as cancer therapy, Alzheimer’s disease treatment or generalized immune stimulation to prevent diverse infections^3^,^27^,^28^,^29^.

## Methods

### Bacterial strains and plasmids

All mutants were created in a *N. meningitidis* strain HB-1, a capsule-deficient derivative of strain H44/76 obtained by transformation with plasmid pMF121, resulting in deletion of the capsular biosynthesis locus including the *galE* gene. *N. meningitidis* strains were grown on GC medium base (Difco) plates supplemented with IsoVitaleX, in a humid atmosphere containing 5% CO_2_ at 37 °C. For liquid culture, strains were grown in 36 mg/mL tryptic soy broth medium (Difco) in a conical flask at 37 °C, shaken at 140 RPM. Required antibiotics were added to plate and liquid cultures (kanamycin 100 μg/ml, chloramphenicol 3 μg/ml). The *lpxL1* and *lpxL2* mutants were obtained by transformation with a linearized pCRII plasmid (Invitrogen) carrying the genes disrupted with a kanamycin resistance cassette described by van der Ley *et al*.^15^ or a pGem-T easy plasmid (Promega) with the *lpxL1* gene that has a deleted section replaced with a chloramphenicol (CAM) cassette. For construction of the *lptA* mutant the gene was amplified by PCR from H44/76, cloned into a pGem-T easy plasmid (Promega) and a kanamycin cassette was inserted at the MunI restriction site. The resulting plasmid was linearized and used for transformation of *N. meningitidis* strain HB-1. *N. meningitidis* derivatives carrying the heterologous genes *pagL, lpxP* and *lpxE* were created using the pEN11 shuttle plasmid previously described for the expression of the *Bordetella bronchiseptica pagL* gene^13^,^30^. To obtain *lpxP* and *lpxE* derivatives, the genes were amplified by PCR from *E. coli* and *B. bronchiseptica*, respectively, and subsequently cloned into pEN11 by replacing the *pagL* gene using restriction enzymes. Expression of heterologous genes cloned in pEN11 was induced by addition of 1 mM isopropyl-β-D-thiogalactopyranoside (IPTG) to the liquid culture medium. Primers are listed in Table 1.

### LPS isolation

LPS from bacterial mutants was extracted with hot phenol-water^31^ and purified further by solid phase extraction (SPE) on reverse phase cartridges. In short, cells from 50 ml of bacterial culture with an OD_600 nm_ of 1.4 (or 100 ml of the *ΔlpxL1-lpxP* mutant grown at 30 °C) were collected by centrifugation at 2,739 × g for 1 h at 20 °C. Then, bacteria were suspended in 20 ml of water and centrifuged at 2,739 × g for 25 min at 20 °C. For hot phenol-water extraction, bacterial pellets were suspended with 4 ml of water, heated to 70 °C, mixed with 3.2 ml of phenol at the same temperature and kept under agitation for 10 min at 70 °C. The aqueous phase was separated from the phenolic phase by centrifugation at 2,739 × g for 15 min at 20 °C. After transferring the aqueous phase to a new vial, the phenolic phase was extracted again by adding 3 ml of water at 70 °C and repeating the extraction procedure. The aqueous phases from two consecutive extractions were pooled (∼6.5 ml) and prepared for SPE by adding 5 ml of 0.356 M triethylammonium acetate (TEAA) pH 7 (solvent A) and 3.8 ml of 2-propanol:water:triethylamine:acetic acid (70:30:0.03:0.01, v/v) pH 8.7 (solvent B). In total, ten LPS extracts each from a different bacterial mutant could be purified simultaneously by SPE on reverse phase Sep-Pak C18 cartridges (1 ml syringe-barrel-type Vac cartridge, 50 mg of C18 resin, Waters) using a 20-position vacuum manifold (Waters). Cartridges were conditioned for SPE by applying consecutively 1 ml of 2-propanol:water:triethylamine:acetic acid (85:15:0.015:0.005, v/v) pH 8.7 (solvent C), 0.07 mM TEAA pH 7 (solvent D) and solvent A under vacuum. Then, samples were split into two aliquots of equal volume and each aliquot was applied into a different cartridge. Next, cartridges were washed once with 1 ml of solvent A and twice with 1 ml of 20% (v/v) solvent B in solvent D. LPS was eluted from the columns by applying 0.6 ml of solvent C. Eluates from the same sample were combined (1.2 ml per sample in total) and dried in a centrifugal vacuum concentrator (Concentrator plus, Eppendorf) at room temperature. LPS concentration in isolated samples was determined by the 3-deoxy-d-*manno*-oct-2-ulosonic acid (Kdo) assay^32^. In addition, the purity and integrity of purified samples were judged by Tricine-SDS-PAGE using 1 mm-thick, 16% precast Novex^®^ mini-gels (Thermo Fisher Scientific Inc.), LPS silver staining^33^ and protein visualization with Imperial^TM^ Protein Stain (Thermo Scientific).

### Mass spectrometry

Electrospray ionization Fourier transform mass spectrometry (ESI-FT-MS) was performed on an LTQ Orbitrap XL instrument (Thermo Scientific) in negative ion mode. LPS samples were dissolved in a mixture of water, 2-propanol and triethylamine (50:50:0.001, by volume) pH 8.5 and infused into the mass spectrometer by static nano-ESI^34^,^35^. The MS instrument was calibrated with a Pierce Negative Ion Calibration Solution (Thermo Scientific) and internally with taurocholic acid following standard procedures provided by the manufacturer (Thermo Scientific). Fragmentation analysis of intact LPS was carried out by in-source collision-induced fragmentation (SID). Y- and B-type fragment ions, corresponding to the lipid A and oligosaccharide moieties of LPS, respectively, were generated by SID at a potential difference of 100 V. Fragment ions are annotated according to the nomenclature of Domon and Costello^36^. Mass spectra were charge-deconvoluted using the Xtract tool of Thermo Xcalibur 3.0 software (Thermo Scientific). All mass values given refer to monoisotopic molecular masses. Proposed LPS compositions are based on the general chemical structure of the L3 immunotype LPS from N. *meningitidis* reported previously^37^,^38^.

### Cell stimulation

Mono Mac 6 cells were seeded at 1 × 10^5^ cells per well in 96 well microtiter plates in 100 μl Iscove’s modified Dulbecco’s medium (IMDM) (Gibco, ThermoFisher scientific) medium supplemented with 100 units/ml penicillin, 100 μg/ml streptomycin, 292 μg/ml l-glutamine (Gibco, ThermoFisher scientific), and 10% fetal calf serum (Gibco, ThermoFisher scientific). Hek blue-hTLR4 cells (Invivogen), a HEK293 cell line stably expressing human TLR4, MD-2 and CD14, were seeded at 3.5 × 10^4^ cells per well in 96-well microtiter plates in 100 μl DMEM (Gibco, ThermoFisher scientific) medium supplemented with 100 units/ml penicillin, 100 μg/ml streptomycin, 292 μg/ml l-glutamine (Gibco, ThermoFisher scientific) and 10% fetal calf serum (Gibco, ThermoFisher scientific). Cells were stimulated with 10-fold serial dilutions of LPS in IMDM (MM6 cells) or DMEM (HEK blue-hTLR4 cells) for 18–20 h at 37 °C in a humid atmosphere containing 5% CO_2_. HEK-blue-hTLR4 cells were also stimulated with serial dilution of heat-inactivated (1 h at 56 °C) whole bacterial cells. Cytokine concentration in the supernatants of MM6 cells was determined by enzyme-linked immunosorbent assay (ELISA). All cytokine (IL-6, IL-1β, IP-10, MCP-1) concentrations were determined using a DUOset ELISA development kit (R&D systems) following the manufacturer’s instructions. To quantify alkaline phosphatase secreted by HEK-blue-hTLR4 cells, 20 μl of the supernatant from each well was added to 200 μl Quanti-blue (Invivogen) and incubated at 37 °C for 2–3 hours. Read out was done on a spectrophotometer at 649 nm. Statistically significant differences were determined by the one-way (alkaline phosphatase secretion) or two-way (cytokine release) ANOVA test by using GraphPad Prism 6.04 statistical software (GraphPad Software, Inc.).

## Additional Information

**How to cite this article**: Zariri, A. *et al*. Modulating endotoxin activity by combinatorial bioengineering of meningococcal lipopolysaccharide. *Sci. Rep.* **6**, 36575; doi: 10.1038/srep36575 (2016).

**Publisher’s note:** Springer Nature remains neutral with regard to jurisdictional claims in published maps and institutional affiliations.

## Supplementary Material

Supplementary Information

## Figures and Tables

**Table 1:** Primers used in this study.

Primer	Sequence (5′-3′)	Source
LptA Fw	GCCTTCCTTTCCCTGTATTC	*N. meningitidis*
LptA Re	GGTGTTCGGACACATATGC	*N. meningitidis*
LpxL1 Fw	CTGATCGGGCAGATACAG	*N. meningitidis*
LpxL1 Re	GTGCGCTACCGCAATAAG	*N. meningitidis*
LpxL2 Fw	AAACAGATACTGCGTCGGAA	*N. meningitidis*
LpxL2 Re	CCCTTTGCGAACCGCCAT	*N. meningitidis*
PagL Fw	ATGCAATTTCTCAAG	*B. bronchiseptica*
PagL Re	TCAGAACTGGTACGT	*B. bronchiseptica*
LpxP Fw	CATATGGCCGCTTACGCAGACAATACAC	*E. coli*
LpxP Re	GACGTCACGCCTGAATGACTTCATTACACC	*E. coli*
LpxE Fw	CATATGATCCGGCCCTCATCCCATTCCC	*B. bronchiseptica*
LpxE Re	TCATGACCCGAAAGGCGCTTCCCTTCAG	*B. bronchiseptica*
