# Supplementary material for: Modulating endotoxin activity by combinatorial bioengineering of meningococcal lipopolysaccharide
Source: Sci Rep. 2016 Nov 14;6:36575. doi: 10.1038/srep36575 (PMC5107901; doi:10.1038/srep36575)
Supplement: Supplementary Information [file srep36575-s1.pdf]

Modulating endotoxin activity by combinatorial bioengineering of meningococcal lipopolysaccharide

Afshin Zariri[1,2], Elder Pupo[1], Elly van Riet[1], Jos P.M. van Putten[2], Peter van der Ley[1]

Supplemental Table 1. Composition of the main ion peaks observed in charge-deconvoluted ESI-FT mass spectra of intact LPS from twelve mutants of *N. meningitidis* (see Fig. 2).

**Bacteria**	**Measured mass (*u*)**	**Proposed LPS composition**		**Calculated mass (*u*)**	**Deviation (*ppm*)**
		**Oligosaccharide**	**Lipid A**		
HB-1	3408.507	PEA$_1$•Hex$_1$•Hep$_2$•HexNAc$_1$•Kdo$_2$•Gly$_1$	P$_3$•PEA$_2$•HexN$_2$•C12$_2$•C12OH$_2$•C14OH$_2$	3408.514	2.2
	3351.488	PEA$_1$•Hex$_1$•Hep$_2$•HexNAc$_1$•Kdo$_2$	P$_3$•PEA$_2$•HexN$_2$•C12$_2$•C12OH$_2$•C14OH$_2$	3351.493	1.5
	3285.501	PEA$_1$•Hex$_1$•Hep$_2$•HexNAc$_1$•Kdo$_2$•Gly$_1$	P$_3$•PEA$_1$•HexN$_2$•C12$_2$•C12OH$_2$•C14OH$_2$	3285.506	1.5
	3228.480	PEA$_1$•Hex$_1$•Hep$_2$•HexNAc$_1$•Kdo$_2$	P$_3$•PEA$_1$•HexN$_2$•C12$_2$•C12OH$_2$•C14OH$_2$	3228.484	1.4
ΔlpxL1	3226.342	PEA$_1$•Hex$_1$•Hep$_2$•HexNAc$_1$•Kdo$_2$•Gly$_1$	P$_3$•PEA$_2$•HexN$_2$•C12$_1$•C12OH$_2$•C14OH$_2$	3226.347	1.7
	3169.324	PEA$_1$•Hex$_1$•Hep$_2$•HexNAc$_1$•Kdo$_2$	P$_3$•PEA$_2$•HexN$_2$•C12$_1$•C12OH$_2$•C14OH$_2$	3169.326	0.6
	3103.336	PEA$_1$•Hex$_1$•Hep$_2$•HexNAc$_1$•Kdo$_2$•Gly$_1$	P$_3$•PEA$_1$•HexN$_2$•C12$_1$•C12OH$_2$•C14OH$_2$	3103.339	0.9
	3046.315	PEA$_1$•Hex$_1$•Hep$_2$•HexNAc$_1$•Kdo$_2$	P$_3$•PEA$_1$•HexN$_2$•C12$_1$•C12OH$_2$•C14OH$_2$	3046.317	0.8
	3388.394	PEA$_1$•Hex$_2$•Hep$_2$•HexNAc$_1$•Kdo$_2$•Gly$_1$	P$_3$•PEA$_2$•HexN$_2$•C12$_1$•C12OH$_2$•C14OH$_2$	3388.400	1.8
	3331.373	PEA$_1$•Hex$_2$•Hep$_2$•HexNAc$_1$•Kdo$_2$	P$_3$•PEA$_2$•HexN$_2$•C12$_1$•C12OH$_2$•C14OH$_2$	3331.379	1.7
ΔlpxL2	3023.367	PEA$_1$•Hex$_1$•Hep$_2$•HexNAc$_1$•Kdo$_2$•Gly$_1$	P$_2$•PEA$_1$•HexN$_2$•C12$_1$•C12OH$_2$•C14OH$_2$	3023.373	1.8
	2966.348	PEA$_1$•Hex$_1$•Hep$_2$•HexNAc$_1$•Kdo$_2$	P$_2$•PEA$_1$•HexN$_2$•C12$_1$•C12OH$_2$•C14OH$_2$	2966.351	1.0
	3185.419	PEA$_1$•Hex$_2$•Hep$_2$•HexNAc$_1$•Kdo$_2$•Gly$_1$	P$_2$•PEA$_1$•HexN$_2$•C12$_1$•C12OH$_2$•C14OH$_2$	3185.425	2.0

Bacteria	Measured mass (*u*)	Proposed LPS composition		Calculated mass (*u*)	Deviation (*ppm*)
		Oligosaccharide	Lipid A		
	3128.398	PEA$_1$•Hex$_2$•Hep$_2$•HexNAc$_1$•Kdo$_2$	P$_2$•PEA$_1$•HexN$_2$•C12$_1$•C12OH$_2$•C14OH$_2$	3128.404	1.9
	2843.340	PEA$_1$•Hex$_1$•Hep$_2$•HexNAc$_1$•Kdo$_2$	P$_2$•HexN$_2$•C12$_1$•C12OH$_2$•C14OH$_2$	2843.343	0.9
	2720.331	Hex$_1$•Hep$_2$•HexNAc$_1$•Kdo$_2$	P$_2$•HexN$_2$•C12$_1$•C12OH$_2$•C14OH$_2$	2720.334	1.1
pagL	3210.345	PEA$_1$•Hex$_1$•Hep$_2$•HexNAc$_1$•Kdo$_2$•Gly$_1$	P$_3$•PEA$_2$•HexN$_2$•C12$_2$•C12OH$_1$•C14OH$_2$	3210.352	2.3
	3232.326*	PEA$_1$•Hex$_1$•Hep$_2$•HexNAc$_1$•Kdo$_2$•Gly$_1$	P$_3$•PEA$_2$•HexN$_2$•C12$_2$•C12OH$_1$•C14OH$_2$	3232.335	2.8
	3153.325	PEA$_1$•Hex$_1$•Hep$_2$•HexNAc$_1$•Kdo$_2$	P$_3$•PEA$_2$•HexN$_2$•C12$_2$•C12OH$_1$•C14OH$_2$	3153.331	1.9
	3175.306*	PEA$_1$•Hex$_1$•Hep$_2$•HexNAc$_1$•Kdo$_2$	P$_3$•PEA$_2$•HexN$_2$•C12$_2$•C12OH$_1$•C14OH$_2$	3175.313	2.4
	3087.338	PEA$_1$•Hex$_1$•Hep$_2$•HexNAc$_1$•Kdo$_2$•Gly$_1$	P$_3$•PEA$_1$•HexN$_2$•C12$_2$•C12OH$_1$•C14OH$_2$	3087.344	1.9
	3109.320*	PEA$_1$•Hex$_1$•Hep$_2$•HexNAc$_1$•Kdo$_2$•Gly$_1$	P$_3$•PEA$_1$•HexN$_2$•C12$_2$•C12OH$_1$•C14OH$_2$	3109.326	2.1
	3030.318	PEA$_1$•Hex$_1$•Hep$_2$•HexNAc$_1$•Kdo$_2$	P$_3$•PEA$_1$•HexN$_2$•C12$_2$•C12OH$_1$•C14OH$_2$	3030.322	1.5
	3052.298*	PEA$_1$•Hex$_1$•Hep$_2$•HexNAc$_1$•Kdo$_2$	P$_3$•PEA$_1$•HexN$_2$•C12$_2$•C12OH$_1$•C14OH$_2$	3052.305	2.3
	2971.161	PEA$_1$•Hex$_1$•Hep$_2$•HexNAc$_1$•Kdo$_2$	P$_3$•PEA$_2$•HexN$_2$•C12$_1$•C12OH$_1$•C14OH$_2$	2971.164	1.0
	2950.352	PEA$_1$•Hex$_1$•Hep$_2$•HexNAc$_1$•Kdo$_2$	P$_2$•PEA$_1$•HexN$_2$•C12$_2$•C12OH$_1$•C14OH$_2$	2950.356	1.4
	2848.152	PEA$_1$•Hex$_1$•Hep$_2$•HexNAc$_1$•Kdo$_2$	P$_3$•PEA$_1$•HexN$_2$•C12$_1$•C12OH$_1$•C14OH$_2$	2848.155	1.2
	3408.505	PEA$_1$•Hex$_1$•Hep$_2$•HexNAc$_1$•Kdo$_2$•Gly$_1$	P$_3$•PEA$_2$•HexN$_2$•C12$_2$•C12OH$_2$•C14OH$_2$	3408.514	2.8
	3351.485	PEA$_1$•Hex$_1$•Hep$_2$•HexNAc$_1$•Kdo$_2$	P$_3$•PEA$_2$•HexN$_2$•C12$_2$•C12OH$_2$•C14OH$_2$	3351.493	2.4
	3372.396	PEA$_1$•Hex$_2$•Hep$_2$•HexNAc$_1$•Kdo$_2$•Gly$_1$	P$_3$•PEA$_2$•HexN$_2$•C12$_2$•C12OH$_1$•C14OH$_2$	3372.405	2.8
	3315.376	PEA$_1$•Hex$_2$•Hep$_2$•HexNAc$_1$•Kdo$_2$	P$_3$•PEA$_2$•HexN$_2$•C12$_2$•C12OH$_1$•C14OH$_2$	3315.384	2.4

Bacteria	Measured mass (*u*)	Proposed LPS composition		Calculated mass (*u*)	Deviation (*ppm*)
		Oligosaccharide	Lipid A		
ΔlpxL1-pagL	3028.180	PEA$_1$•Hex$_1$•Hep$_2$•HexNAc$_1$•Kdo$_2$•Gly$_1$	P$_3$•PEA$_2$•HexN$_2$•C12$_1$•C12OH$_1$•C14OH$_2$	3028.185	1.8
	2971.160	PEA$_1$•Hex$_1$•Hep$_2$•HexNAc$_1$•Kdo$_2$	P$_3$•PEA$_2$•HexN$_2$•C12$_1$•C12OH$_1$•C14OH$_2$	2971.164	1.3
	2905.173	PEA$_1$•Hex$_1$•Hep$_2$•HexNAc$_1$•Kdo$_2$•Gly$_1$	P$_3$•PEA$_1$•HexN$_2$•C12$_1$•C12OH$_1$•C14OH$_2$	2905.177	1.3
	2848.152	PEA$_1$•Hex$_1$•Hep$_2$•HexNAc$_1$•Kdo$_2$	P$_3$•PEA$_1$•HexN$_2$•C12$_1$•C12OH$_1$•C14OH$_2$	2848.155	1.2
	3226.339	PEA$_1$•Hex$_1$•Hep$_2$•HexNAc$_1$•Kdo$_2$•Gly$_1$	P$_3$•PEA$_2$•HexN$_2$•C12$_1$•C12OH$_2$•C14OH$_2$	3226.347	2.6
	3169.319	PEA$_1$•Hex$_1$•Hep$_2$•HexNAc$_1$•Kdo$_2$	P$_3$•PEA$_2$•HexN$_2$•C12$_1$•C12OH$_2$•C14OH$_2$	3169.326	2.2
	3190.232	PEA$_1$•Hex$_2$•Hep$_2$•HexNAc$_1$•Kdo$_2$•Gly$_1$	P$_3$•PEA$_2$•HexN$_2$•C12$_1$•C12OH$_1$•C14OH$_2$	3190.238	2.0
	3133.210	PEA$_1$•Hex$_2$•Hep$_2$•HexNAc$_1$•Kdo$_2$	P$_3$•PEA$_2$•HexN$_2$•C12$_1$•C12OH$_1$•C14OH$_2$	3133.217	2.2
	3103.331	PEA$_1$•Hex$_1$•Hep$_2$•HexNAc$_1$•Kdo$_2$•Gly$_1$	P$_3$•PEA$_1$•HexN$_2$•C12$_1$•C12OH$_2$•C14OH$_2$	3103.339	2.5
ΔlpxL2-pagL	2825.206	PEA$_1$•Hex$_1$•Hep$_2$•HexNAc$_1$•Kdo$_2$•Gly$_1$	P$_2$•PEA$_1$•HexN$_2$•C12$_1$•C12OH$_1$•C14OH$_2$	2825.211	1.6
	2768.187	PEA$_1$•Hex$_1$•Hep$_2$•HexNAc$_1$•Kdo$_2$	P$_2$•PEA$_1$•HexN$_2$•C12$_1$•C12OH$_1$•C14OH$_2$	2768.189	0.8
	2987.257	PEA$_1$•Hex$_2$•Hep$_2$•HexNAc$_1$•Kdo$_2$•Gly$_1$	P$_2$•PEA$_1$•HexN$_2$•C12$_1$•C12OH$_1$•C14OH$_2$	2987.263	2.1
	2930.236	PEA$_1$•Hex$_2$•Hep$_2$•HexNAc$_1$•Kdo$_2$	P$_2$•PEA$_1$•HexN$_2$•C12$_1$•C12OH$_1$•C14OH$_2$	2930.242	2.0
	2966.344	PEA$_1$•Hex$_1$•Hep$_2$•HexNAc$_1$•Kdo$_2$	P$_2$•PEA$_1$•HexN$_2$•C12$_1$•C12OH$_2$•C14OH$_2$	2966.351	2.4
	2548.127	PEA$_1$•Hex$_1$•Hep$_2$•HexNAc$_1$•Kdo$_1$	P$_2$•PEA$_1$•HexN$_2$•C12$_1$•C12OH$_1$•C14OH$_2$	2548.131	1.5
	2645.178	PEA$_1$•Hex$_1$•Hep$_2$•HexNAc$_1$•Kdo$_2$	P$_2$•HexN$_2$•C12$_1$•C12OH$_1$•C14OH$_2$	2645.181	1.0
	2720.331	Hex$_1$•Hep$_2$•HexNAc$_1$•Kdo$_2$	P$_2$•HexN$_2$•C12$_1$•C12OH$_2$•C14OH$_2$	2720.334	1.1
ΔlpxL1-lpxP 30°C	3046.315	PEA$_1$•Hex$_1$•Hep$_2$•HexNAc$_1$•Kdo$_2$	P$_3$•PEA$_1$•HexN$_2$•C12$_1$•C12OH$_2$•C14OH$_2$	3046.317	0.8

Bacteria	Measured mass (*u*)	Proposed LPS composition		Calculated mass (*u*)	Deviation (*ppm*)
		Oligosaccharide	Lipid A		
	3103.333	PEA$_1$•Hex$_1$•Hep$_2$•HexNAc$_1$•Kdo$_2$•Gly$_1$	P$_3$•PEA$_1$•HexN$_2$•C12$_1$•C12OH$_2$•C14OH$_2$	3103.339	1.9
	3169.322	PEA$_1$•Hex$_1$•Hep$_2$•HexNAc$_1$•Kdo$_2$	P$_3$•PEA$_2$•HexN$_2$•C12$_1$•C12OH$_2$•C14OH$_2$	3169.326	1.2
	3226.340	PEA$_1$•Hex$_1$•Hep$_2$•HexNAc$_1$•Kdo$_2$•Gly$_1$	P$_3$•PEA$_2$•HexN$_2$•C12$_1$•C12OH$_2$•C14OH$_2$	3226.347	2.3
	3208.364	PEA$_1$•Hex$_2$•Hep$_2$•HexNAc$_1$•Kdo$_2$	P$_3$•PEA$_1$•HexN$_2$•C12$_1$•C12OH$_2$•C14OH$_2$	3208.370	1.9
	3265.382	PEA$_1$•Hex$_2$•Hep$_2$•HexNAc$_1$•Kdo$_2$•Gly$_1$	P$_3$•PEA$_1$•HexN$_2$•C12$_1$•C12OH$_2$•C14OH$_2$	3265.392	3.0
	3331.372	PEA$_1$•Hex$_2$•Hep$_2$•HexNAc$_1$•Kdo$_2$	P$_3$•PEA$_2$•HexN$_2$•C12$_1$•C12OH$_2$•C14OH$_2$	3331.379	2.0
	3388.391	PEA$_1$•Hex$_2$•Hep$_2$•HexNAc$_1$•Kdo$_2$•Gly$_1$	P$_3$•PEA$_2$•HexN$_2$•C12$_1$•C12OH$_2$•C14OH$_2$	3388.400	2.7
	3282.524	PEA$_1$•Hex$_1$•Hep$_2$•HexNAc$_1$•Kdo$_2$	P$_3$•PEA$_1$•HexN$_2$•C12$_1$•C12OH$_2$•C14OH$_2$.C16:1$_1$	3282.531	2.3
	3339.543	PEA$_1$•Hex$_1$•Hep$_2$•HexNAc$_1$•Kdo$_2$•Gly$_1$	P$_3$•PEA$_1$•HexN$_2$•C12$_1$•C12OH$_2$•C14OH$_2$.C16:1$_1$	3339.553	3.0
	3405.533	PEA$_1$•Hex$_1$•Hep$_2$•HexNAc$_1$•Kdo$_2$	P$_3$•PEA$_2$•HexN$_2$•C12$_1$•C12OH$_2$•C14OH$_2$•C16:1$_1$	3405.540	2.0
	3462.553	PEA$_1$•Hex$_1$•Hep$_2$•HexNAc$_1$•Kdo$_2$•Gly$_1$	P$_3$•PEA$_2$•HexN$_2$•C12$_1$•C12OH$_2$•C14OH$_2$.C16:1$_1$	3462.561	2.4
	3444.574	PEA$_1$•Hex$_2$•Hep$_2$•HexNAc$_1$•Kdo$_2$	P$_3$•PEA$_1$•HexN$_2$•C12$_1$•C12OH$_2$•C14OH$_2$.C16:1$_1$	3444.584	3.0
	3567.585	PEA$_1$•Hex$_2$•Hep$_2$•HexNAc$_1$•Kdo$_2$	P$_3$•PEA$_2$•HexN$_2$•C12$_1$•C12OH$_2$•C14OH$_2$•C16:1$_1$	3567.593	2.2
	3624.606	PEA$_1$•Hex$_2$•Hep$_2$•HexNAc$_1$•Kdo$_2$•Gly$_1$	P$_3$•PEA$_2$•HexN$_2$•C12$_1$•C12OH$_2$•C14OH$_2$.C16:1$_1$	3624.614	2.3
ΔlpxL1-lpxP 25°C	3046.314	PEA$_1$•Hex$_1$•Hep$_2$•HexNAc$_1$•Kdo$_2$	P$_3$•PEA$_1$•HexN$_2$•C12$_1$•C12OH$_2$•C14OH$_2$	3046.317	1.1
	3103.333	PEA$_1$•Hex$_1$•Hep$_2$•HexNAc$_1$•Kdo$_2$•Gly$_1$	P$_3$•PEA$_1$•HexN$_2$•C12$_1$•C12OH$_2$•C14OH$_2$	3103.339	1.9
	3169.321	PEA$_1$•Hex$_1$•Hep$_2$•HexNAc$_1$•Kdo$_2$	P$_3$•PEA$_2$•HexN$_2$•C12$_1$•C12OH$_2$•C14OH$_2$	3169.326	1.6
	3226.341	PEA$_1$•Hex$_1$•Hep$_2$•HexNAc$_1$•Kdo$_2$•Gly$_1$	P$_3$•PEA$_2$•HexN$_2$•C12$_1$•C12OH$_2$•C14OH$_2$	3226.347	2.0

Bacteria	Measured mass (*u*)	Proposed LPS composition		Calculated mass (*u*)	Deviation (*ppm*)
		**Oligosaccharide**	**Lipid A**		
	3208.366	PEA$_1$•Hex$_2$•Hep$_2$•HexNAc$_1$•Kdo$_2$	P$_3$•PEA$_1$•HexN$_2$•C12$_1$•C12OH$_2$•C14OH$_2$	3208.370	1.3
	3265.386	PEA$_1$•Hex$_2$•Hep$_2$•HexNAc$_1$•Kdo$_2$•Gly$_1$	P$_3$•PEA$_1$•HexN$_2$•C12$_1$•C12OH$_2$•C14OH$_2$	3265.392	1.7
	3331.373	PEA$_1$•Hex$_2$•Hep$_2$•HexNAc$_1$•Kdo$_2$	P$_3$•PEA$_2$•HexN$_2$•C12$_1$•C12OH$_2$•C14OH$_2$	3331.379	1.7
	3388.393	PEA$_1$•Hex$_2$•Hep$_2$•HexNAc$_1$•Kdo$_2$•Gly$_1$	P$_3$•PEA$_2$•HexN$_2$•C12$_1$•C12OH$_2$•C14OH$_2$	3388.400	2.1
	3254.494	PEA$_1$•Hex$_1$•Hep$_2$•HexNAc$_1$•Kdo$_2$	P$_3$•PEA$_1$•HexN$_2$•C12$_1$•C12OH$_2$•C14OH$_2$•C16:1$_1$ -C2H4	3254.500	1.9
	3282.526	PEA$_1$•Hex$_1$•Hep$_2$•HexNAc$_1$•Kdo$_2$	P$_3$•PEA$_1$•HexN$_2$•C12$_1$•C12OH$_2$•C14OH$_2$•C16:1$_1$	3282.531	1.6
	3311.516	PEA$_1$•Hex$_1$•Hep$_2$•HexNAc$_1$•Kdo$_2$•Gly$_1$	P$_3$•PEA$_1$•HexN$_2$•C12$_1$•C12OH$_2$•C14OH$_2$•C16:1$_1$ -C2H4	3311.522	1.7
	3339.546	PEA$_1$•Hex$_1$•Hep$_2$•HexNAc$_1$•Kdo$_2$•Gly$_1$	P$_3$•PEA$_1$•HexN$_2$•C12$_1$•C12OH$_2$•C14OH$_2$•C16:1$_1$	3339.553	2.1
	3377.502	PEA$_1$•Hex$_1$•Hep$_2$•HexNAc$_1$•Kdo$_2$	P$_3$•PEA$_2$•HexN$_2$•C12$_1$•C12OH$_2$•C14OH$_2$•C16:1$_1$ -C2H4	3377.509	2.0
	3405.535	PEA$_1$•Hex$_1$•Hep$_2$•HexNAc$_1$•Kdo$_2$	P$_3$•PEA$_2$•HexN$_2$•C12$_1$•C12OH$_2$•C14OH$_2$•C16:1$_1$	3405.540	1.4
	3434.522	PEA$_1$•Hex$_1$•Hep$_2$•HexNAc$_1$•Kdo$_2$•Gly$_1$	P$_3$•PEA$_2$•HexN$_2$•C12$_1$•C12OH$_2$•C14OH$_2$•C16:1$_1$ -C2H4	3434.530	2.4
	3462.554	PEA$_1$•Hex$_1$•Hep$_2$•HexNAc$_1$•Kdo$_2$•Gly$_1$	P$_3$•PEA$_2$•HexN$_2$•C12$_1$•C12OH$_2$•C14OH$_2$•C16:1$_1$	3462.561	2.1
	3444.575	PEA$_1$•Hex$_2$•Hep$_2$•HexNAc$_1$•Kdo$_2$	P$_3$•PEA$_1$•HexN$_2$•C12$_1$•C12OH$_2$•C14OH$_2$•C16:1$_1$	3444.584	2.7
	3501.596	PEA$_1$•Hex$_2$•Hep$_2$•HexNAc$_1$•Kdo$_2$•Gly$_1$	P$_3$•PEA$_1$•HexN$_2$•C12$_1$•C12OH$_2$•C14OH$_2$•C16:1$_1$	3501.606	2.8
	3567.586	PEA$_1$•Hex$_2$•Hep$_2$•HexNAc$_1$•Kdo$_2$	P$_3$•PEA$_2$•HexN$_2$•C12$_1$•C12OH$_2$•C14OH$_2$•C16:1$_1$	3567.593	1.9
	3624.608	PEA$_1$•Hex$_2$•Hep$_2$•HexNAc$_1$•Kdo$_2$•Gly$_1$	P$_3$•PEA$_2$•HexN$_2$•C12$_1$•C12OH$_2$•C14OH$_2$•C16:1$_1$	3624.614	1.7
ΔlptA	3162.489	PEA$_1$•Hex$_1$•Hep$_2$•HexNAc$_1$•Kdo$_2$•Gly$_1$	P$_3$•HexN$_2$•C12$_2$•C12OH$_2$•C14OH$_2$	3162.497	2.7
	3184.471*	PEA$_1$•Hex$_1$•Hep$_2$•HexNAc$_1$•Kdo$_2$•Gly$_1$	P$_3$•HexN$_2$•C12$_2$•C12OH$_2$•C14OH$_2$	3184.480	2.8

Bacteria	Measured mass (*u*)	Proposed LPS composition: Oligosaccharide	Proposed LPS composition: Lipid A	Calculated mass (*u*)	Deviation (*ppm*)
	3105.471	$PEA_1 \cdot Hex_1 \cdot Hep_2 \cdot HexNAc_1 \cdot Kdo_2$	$P_3 \cdot HexN_2 \cdot C12_2 \cdot C12OH_2 \cdot C14OH_2$	3105.476	1.6
	3127.452*	$PEA_1 \cdot Hex_1 \cdot Hep_2 \cdot HexNAc_1 \cdot Kdo_2$	$P_3 \cdot HexN_2 \cdot C12_2 \cdot C12OH_2 \cdot C14OH_2$	3127.458	2.0
	3025.506	$PEA_1 \cdot Hex_1 \cdot Hep_2 \cdot HexNAc_1 \cdot Kdo_2$	$P_2 \cdot HexN_2 \cdot C12_2 \cdot C12OH_2 \cdot C14OH_2$	3025.510	1.2
	3324.541	$PEA_1 \cdot Hex_2 \cdot Hep_2 \cdot HexNAc_1 \cdot Kdo_2 \cdot Gly_1$	$P_3 \cdot HexN_2 \cdot C12_2 \cdot C12OH_2 \cdot C14OH_2$	3324.550	2.8
	3267.521	$PEA_1 \cdot Hex_2 \cdot Hep_2 \cdot HexNAc_1 \cdot Kdo_2$	$P_3 \cdot HexN_2 \cdot C12_2 \cdot C12OH_2 \cdot C14OH_2$	3267.529	2.4
ΔlptA-ΔlpxL1	2980.324	$PEA_1 \cdot Hex_1 \cdot Hep_2 \cdot HexNAc_1 \cdot Kdo_2 \cdot Gly_1$	$P_3 \cdot HexN_2 \cdot C12_1 \cdot C12OH_2 \cdot C14OH_2$	2980.330	2.1
	2923.307	$PEA_1 \cdot Hex_1 \cdot Hep_2 \cdot HexNAc_1 \cdot Kdo_2$	$P_3 \cdot HexN_2 \cdot C12_1 \cdot C12OH_2 \cdot C14OH_2$	2923.309	0.6
	3142.375	$PEA_1 \cdot Hex_2 \cdot Hep_2 \cdot HexNAc_1 \cdot Kdo_2 \cdot Gly_1$	$P_3 \cdot HexN_2 \cdot C12_1 \cdot C12OH_2 \cdot C14OH_2$	3142.383	2.6
	3085.354	$PEA_1 \cdot Hex_2 \cdot Hep_2 \cdot HexNAc_1 \cdot Kdo_2$	$P_3 \cdot HexN_2 \cdot C12_1 \cdot C12OH_2 \cdot C14OH_2$	3085.362	2.5
ΔlptA-pagL	2964.328	$PEA_1 \cdot Hex_1 \cdot Hep_2 \cdot HexNAc_1 \cdot Kdo_2 \cdot Gly_1$	$P_3 \cdot HexN_2 \cdot C12_2 \cdot C12OH_1 \cdot C14OH_2$	2964.335	2.5
	2986.309*	$PEA_1 \cdot Hex_1 \cdot Hep_2 \cdot HexNAc_1 \cdot Kdo_2 \cdot Gly_1$	$P_3 \cdot HexN_2 \cdot C12_2 \cdot C12OH_1 \cdot C14OH_2$	2986.318	3.0
	2907.311	$PEA_1 \cdot Hex_1 \cdot Hep_2 \cdot HexNAc_1 \cdot Kdo_2$	$P_3 \cdot HexN_2 \cdot C12_2 \cdot C12OH_1 \cdot C14OH_2$	2907.314	1.0
	2929.290*	$PEA_1 \cdot Hex_1 \cdot Hep_2 \cdot HexNAc_1 \cdot Kdo_2$	$P_3 \cdot HexN_2 \cdot C12_2 \cdot C12OH_1 \cdot C14OH_2$	2929.296	2.2
	3069.359	$PEA_1 \cdot Hex_2 \cdot Hep_2 \cdot HexNAc_1 \cdot Kdo_2$	$P_3 \cdot HexN_2 \cdot C12_2 \cdot C12OH_1 \cdot C14OH_2$	3069.367	2.5
	3105.468	$PEA_1 \cdot Hex_1 \cdot Hep_2 \cdot HexNAc_1 \cdot Kdo_2$	$P_3 \cdot HexN_2 \cdot C12_2 \cdot C12OH_2 \cdot C14OH_2$	3105.476	2.6
	3126.379	$PEA_1 \cdot Hex_2 \cdot Hep_2 \cdot HexNAc_1 \cdot Kdo_2 \cdot Gly_1$	$P_3 \cdot HexN_2 \cdot C12_2 \cdot C12OH_1 \cdot C14OH_2$	3126.388	3.0
	3162.488	$PEA_1 \cdot Hex_1 \cdot Hep_2 \cdot HexNAc_1 \cdot Kdo_2 \cdot Gly_1$	$P_3 \cdot HexN_2 \cdot C12_2 \cdot C12OH_2 \cdot C14OH_2$	3162.497	3.0
	2725.144	$PEA_1 \cdot Hex_1 \cdot Hep_2 \cdot HexNAc_1 \cdot Kdo_2$	$P_3 \cdot HexN_2 \cdot C12_1 \cdot C12OH_1 \cdot C14OH_2$	2725.147	1.1

Bacteria	Measured mass (*u*)	Proposed LPS composition		Calculated mass (*u*)	Deviation (*ppm*)
		Oligosaccharide	Lipid A		
	2782.165	PEA$_1$•Hex$_1$•Hep$_2$•HexNAc$_1$•Kdo$_2$•Gly$_1$	P$_3$•HexN$_2$•C12$_1$•C12OH$_1$•C14OH$_2$	2782.168	1.2
	2827.344	PEA$_1$•Hex$_1$•Hep$_2$•HexNAc$_1$•Kdo$_2$	P$_2$•HexN$_2$•C12$_2$•C12OH$_1$•C14OH$_2$	2827.348	1.3
	2687.254	PEA$_1$•Hex$_1$•Hep$_2$•HexNAc$_1$•Kdo$_1$	P$_3$•HexN$_2$•C12$_2$•C12OH$_1$•C14OH$_2$	2687.256	0.6
ΔlptA-lpxE	3082.525	PEA$_1$•Hex$_1$•Hep$_2$•HexNAc$_1$•Kdo$_2$•Gly$_1$	P$_2$•HexN$_2$•C12$_2$•C12OH$_2$•C14OH$_2$	3082.531	2.0
	3104.507*	PEA$_1$•Hex$_1$•Hep$_2$•HexNAc$_1$•Kdo$_2$•Gly$_1$	P$_2$•HexN$_2$•C12$_2$•C12OH$_2$•C14OH$_2$	3104.514	2.1
	3025.508	PEA$_1$•Hex$_1$•Hep$_2$•HexNAc$_1$•Kdo$_2$	P$_2$•HexN$_2$•C12$_2$•C12OH$_2$•C14OH$_2$	3025.510	0.5
	3047.488*	PEA$_1$•Hex$_1$•Hep$_2$•HexNAc$_1$•Kdo$_2$	P$_2$•HexN$_2$•C12$_2$•C12OH$_2$•C14OH$_2$	3047.492	1.3
	3069.468**	PEA$_1$•Hex$_1$•Hep$_2$•HexNAc$_1$•Kdo$_2$	P$_2$•HexN$_2$•C12$_2$•C12OH$_2$•C14OH$_2$	3069.475	2.1
	3244.576	PEA$_1$•Hex$_2$•Hep$_2$•HexNAc$_1$•Kdo$_2$•Gly$_1$	P$_2$•HexN$_2$•C12$_2$•C12OH$_2$•C14OH$_2$	3244.584	2.4
	3187.556	PEA$_1$•Hex$_2$•Hep$_2$•HexNAc$_1$•Kdo$_2$	P$_2$•HexN$_2$•C12$_2$•C12OH$_2$•C14OH$_2$	3187.562	2.0
	2805.449	PEA$_1$•Hex$_1$•Hep$_2$•HexNAc$_1$•Kdo$_1$	P$_2$•HexN$_2$•C12$_2$•C12OH$_2$•C14OH$_2$	2805.451	0.8

(*) Monosodium adduct. (**) Disodium adduct. **Abbreviations:** Kdo, 3-deoxy-D-*manno*-oct-2-ulosonic acid; Hep, L-*glycero*-D-*manno*-heptose; Hex, hexose; HexNAc, *N*-acetylhexosamine; Gly, glycine; PEA, phosphoethanolamine; P, phosphate; C12OH, 3-hydroxy-dodecanoic acid; C14OH, 3-hydroxy-tetradecanoic acid; C12, dodecanoic acid; C16:1, 9-hexadecenoic acid

Supplemental Table 2. Proposed compositions for charge-deconvoluted fragment ion peaks obtained by in-source collision-induced dissociation ESI-FT MS of LPS.

Bacteria	Fragment ion type[a)]	Measured mass (*u*)	Proposed LPS composition		Calculated mass (*u*)	Deviation (*ppm*)
			Oligosaccharide	Lipid A		
HB-1	B	1369.404	PE$_1$•Hex$_1$•Hep$_2$•HexNAc$_1$•Kdo$_2$•Gly$_1$		1369.406	1.1
	B-Kdo-CO2	1105.355	PE$_1$•Hex$_1$•Hep$_2$•HexNAc$_1$•Kdo$_2$•Gly$_1$		1105.357	2.2
	B-Kdo-CO2	1048.334	PE$_1$•Hex$_1$•Hep$_2$•HexNAc$_1$•Kdo$_2$		1048.336	1.9
	Y	1916.098		P$_3$•PE$_1$•HexN$_2$•C12$_2$•C12OH$_2$•C14OH$_2$	1916.100	1.2
	Y	2039.106		P$_3$•PE$_2$•HexN$_2$•C12$_2$•C12OH$_2$•C14OH$_2$	2039.109	1.4
ΔlpxL1	B	1369.405	PE$_1$•Hex$_1$•Hep$_2$•HexNAc$_1$•Kdo$_2$•Gly$_1$		1369.406	0.4
	B-Kdo-CO2	1105.356	PE$_1$•Hex$_1$•Hep$_2$•HexNAc$_1$•Kdo$_2$•Gly$_1$		1105.357	1.3
	B-Kdo-CO2	1048.335	PE$_1$•Hex$_1$•Hep$_2$•HexNAc$_1$•Kdo$_2$		1048.336	0.9
	Y	1733.933		P$_3$•PE$_1$•HexN$_2$•C12$_1$•C12OH$_2$•C14OH$_2$	1733.933	0.2
	Y	1856.942		P$_3$•PE$_2$•HexN$_2$•C12$_1$•C12OH$_2$•C14OH$_2$	1856.942	0.1
ΔlpxL2	B	1369.404	PE$_1$•Hex$_1$•Hep$_2$•HexNAc$_1$•Kdo$_2$•Gly$_1$		1369.406	1.1
	B-Kdo-CO2	1105.356	PE$_1$•Hex$_1$•Hep$_2$•HexNAc$_1$•Kdo$_2$•Gly$_1$		1105.357	1.3
	B-Kdo-CO2	1048.335	PE$_1$•Hex$_1$•Hep$_2$•HexNAc$_1$•Kdo$_2$		1048.336	0.9
	B-Kdo-CO2	1267.409	PE$_1$•Hex$_2$•Hep$_2$•HexNAc$_1$•Kdo$_2$•Gly$_1$		1267.410	1.0
	B-Kdo-CO2	1210.387	PE$_1$•Hex$_2$•Hep$_2$•HexNAc$_1$•Kdo$_2$		1210.389	1.5

Bacteria	Fragment ion type[a)]	Measured mass (*u*)	Proposed LPS composition		Calculated mass (*u*)	Deviation (*ppm*)
			Oligosaccharide	Lipid A		
	Y	1530.957		P$_2$•HexN$_2$•C12$_1$•C12OH$_2$•C14OH$_2$	1530.958	0.9
	Y	1653.965		P$_2$•PE$_1$•HexN$_2$•C12$_1$•C12OH$_2$•C14OH$_2$	1653.967	1.2
pagL	B	1369.404	PE$_1$•Hex$_1$•Hep$_2$•HexNAc$_1$•Kdo$_2$•Gly$_1$		1369.406	1.1
	B	1312.381	PE$_1$•Hex$_1$•Hep$_2$•HexNAc$_1$•Kdo$_2$		1312.384	2.4
	B-Kdo-CO2	1105.356	PE$_1$•Hex$_1$•Hep$_2$•HexNAc$_1$•Kdo$_2$•Gly$_1$		1105.357	1.3
	B-Kdo-CO2	1048.335	PE$_1$•Hex$_1$•Hep$_2$•HexNAc$_1$•Kdo$_2$		1048.336	0.9
	B-Kdo-CO2	1210.387	PE$_1$•Hex$_2$•Hep$_2$•HexNAc$_1$•Kdo$_2$		1210.389	1.5
	Y	1717.937		P$_3$•PE$_1$•HexN$_2$•C12$_2$•C12OH$_1$•C14OH$_2$	1717.938	0.8
	Y	1840.946		P$_3$•PE$_2$•HexN$_2$•C12$_2$•C12OH$_1$•C14OH$_2$	1840.947	0.5
ΔlpxL1-pagL	B	1369.404	PE$_1$•Hex$_1$•Hep$_2$•HexNAc$_1$•Kdo$_2$•Gly$_1$		1369.406	1.1
	B-Kdo-CO2	1105.356	PE$_1$•Hex$_1$•Hep$_2$•HexNAc$_1$•Kdo$_2$•Gly$_1$		1105.357	1.3
	B-Kdo-CO2	1048.335	PE$_1$•Hex$_1$•Hep$_2$•HexNAc$_1$•Kdo$_2$		1048.336	0.9
	B-Kdo-CO2	1267.409	PE$_1$•Hex$_2$•Hep$_2$•HexNAc$_1$•Kdo$_2$•Gly$_1$		1267.410	1.0
	B-Kdo-CO2	1210.387	PE$_1$•Hex$_2$•Hep$_2$•HexNAc$_1$•Kdo$_2$		1210.389	1.5
	Y	1535.77		P$_3$•PE$_1$•HexN$_2$•C12$_1$•C12OH$_1$•C14OH$_2$	1535.771	0.8
	Y	1658.779		P$_3$•PE$_2$•HexN$_2$•C12$_1$•C12OH$_1$•C14OH$_2$	1658.780	0.5
	Y	1856.943		P$_3$•PE$_2$•HexN$_2$•C12$_1$•C12OH$_2$•C14OH$_2$	1856.942	0.6
	Y	1733.933		P$_3$•PE$_1$•HexN$_2$•C12$_1$•C12OH$_2$•C14OH$_2$	1733.933	0.2

Bacteria	Fragment ion type[a)]	Measured mass (*u*)	Proposed LPS composition		Calculated mass (*u*)	Deviation (*ppm*)
			Oligosaccharide	Lipid A		
ΔlpxL2-pagL	B-Kdo-CO2	1105.355	PE1•Hex1•Hep2•HexNAc1•Kdo2•Gly1		1105.357	2.2
	B-Kdo-CO2	1048.334	PE1•Hex1•Hep2•HexNAc1•Kdo2		1048.336	1.9
	B-Kdo-CO2	1267.408	PE1•Hex2•Hep2•HexNAc1•Kdo2•Gly1		1267.410	1.8
	B-Kdo-CO2	1210.387	PE1•Hex2•Hep2•HexNAc1•Kdo2		1210.389	1.5
	Y	1455.802		P2•PE1•HexN2•C121•C12OH1•C14OH2	1455.805	2.0
	Y	1653.964		P2•PE1•HexN2•C121•C12OH2•C14OH2	1653.967	1.8
	Y	1530.955		P2•HexN2•C121•C12OH2•C14OH2	1530.958	2.2
ΔlpxL1-LpxP 30°C	B	1369.403	PE1•Hex1•Hep2•HexNAc1•Kdo2•Gly1		1369.406	1.9
	B-Kdo-CO2	1105.356	PE1•Hex1•Hep2•HexNAc1•Kdo2•Gly1		1105.357	1.3
	B-Kdo-CO2	1048.335	PE1•Hex1•Hep2•HexNAc1•Kdo2		1048.336	0.9
	B-Kdo-CO2	1267.408	PE1•Hex2•Hep2•HexNAc1•Kdo2•Gly1		1267.410	1.8
	B-Kdo-CO2	1210.387	PE1•Hex2•Hep2•HexNAc1•Kdo2		1210.389	1.5
	Y	1856.941		P3•PE2•HexN2•C121•C12OH2•C14OH2	1856.942	0.4
	Y	1733.932		P3•PE1•HexN2•C121•C12OH2•C14OH2	1733.933	0.7
	Y-P	1653.965		P3•PE1•HexN2•C121•C12OH2•C14OH2	1653.967	1.2
	Y-2P-H2O	1555.988		P3•PE1•HexN2•C121•C12OH2•C14OH2	1555.990	1.3
	Y	2093.153		P3•PE2•HexN2•C121•C12OH2•C14OH2•C16:11	2093.156	1.4
	Y	1970.147		P3•PE1•HexN2•C121•C12OH2•C14OH2•C16:11	1970.147	0.2

Bacteria	Fragment ion type[a)]	Measured mass (*u*)	Proposed LPS composition		Calculated mass (*u*)	Deviation (*ppm*)
			**Oligosaccharide**	**Lipid A**		
	Y-P	1890.182		P$_3$•PE$_1$•HexN$_2$•C12$_1$•C12OH$_2$•C14OH$_2$•C16:1$_1$	1890.181	0.5
	Y-2P-H2O	1792.202		P$_3$•PE$_1$•HexN$_2$•C12$_1$•C12OH$_2$•C14OH$_2$•C16:1$_1$	1792.204	1.2
ΔlpxL1-LpxP 25°C	B	1369.404	PE$_1$•Hex$_1$•Hep$_2$•HexNAc$_1$•Kdo$_2$•Gly$_1$		1369.406	1.1
	B	1312.379	PE$_1$•Hex$_1$•Hep$_2$•HexNAc$_1$•Kdo$_2$		1312.384	3.9
	B-Kdo-CO2	1105.356	PE$_1$•Hex$_1$•Hep$_2$•HexNAc$_1$•Kdo$_2$•Gly$_1$		1105.357	1.3
	B-Kdo-CO2	1048.335	PE$_1$•Hex$_1$•Hep$_2$•HexNAc$_1$•Kdo$_2$		1048.336	0.9
	B-Kdo-CO2	1267.409	PE$_1$•Hex$_2$•Hep$_2$•HexNAc$_1$•Kdo$_2$•Gly$_1$		1267.410	1.0
	B-Kdo-CO2	1210.387	PE$_1$•Hex$_2$•Hep$_2$•HexNAc$_1$•Kdo$_2$		1210.389	1.5
	Y	1856.941		P$_3$•PE$_2$•HexN$_2$•C12$_1$•C12OH$_2$•C14OH$_2$	1856.942	0.4
	Y	1733.932		P$_3$•PE$_1$•HexN$_2$•C12$_1$•C12OH$_2$•C14OH$_2$	1733.933	0.7
	Y-P	1653.965		P$_3$•PE$_1$•HexN$_2$•C12$_1$•C12OH$_2$•C14OH$_2$	1653.967	1.2
	Y-2P-H2O	1555.988		P$_3$•PE$_1$•HexN$_2$•C12$_1$•C12OH$_2$•C14OH$_2$	1555.990	1.3
	Y	2093.152		P$_3$•PE$_2$•HexN$_2$•C12$_1$•C12OH$_2$•C14OH$_2$•C16:1$_1$	2093.156	1.8
	Y	1970.147		P$_3$•PE$_1$•HexN$_2$•C12$_1$•C12OH$_2$•C14OH$_2$•C16:1$_1$	1970.147	0.2
	Y-P	1890.18		P$_3$•PE$_1$•HexN$_2$•C12$_1$•C12OH$_2$•C14OH$_2$•C16:1$_1$	1890.181	0.5
	Y-2P-H2O	1792.203		P$_3$•PE$_1$•HexN$_2$•C12$_1$•C12OH$_2$•C14OH$_2$•C16:1$_1$	1792.204	0.6
ΔlptA	B	1369.404	PE$_1$•Hex$_1$•Hep$_2$•HexNAc$_1$•Kdo$_2$•Gly$_1$		1369.406	1.1
	B	1312.38	PE$_1$•Hex$_1$•Hep$_2$•HexNAc$_1$•Kdo$_2$		1312.384	3.1

Bacteria	Fragment ion type[a)]	Measured mass (*u*)	Proposed LPS composition		Calculated mass (*u*)	Deviation (*ppm*)
			**Oligosaccharide**	**Lipid A**		
	B-Kdo-CO2	1105.356	PE$_1$•Hex$_1$•Hep$_2$•HexNAc$_1$•Kdo$_2$•Gly$_1$		1105.357	1.3
	B-Kdo-CO2	1048.335	PE$_1$•Hex$_1$•Hep$_2$•HexNAc$_1$•Kdo$_2$		1048.336	0.9
	B-Kdo-CO2	1267.408	PE$_1$•Hex$_2$•Hep$_2$•HexNAc$_1$•Kdo$_2$•Gly$_1$		1267.410	1.8
	B-Kdo-CO2	1210.387	PE$_1$•Hex$_2$•Hep$_2$•HexNAc$_1$•Kdo$_2$		1210.389	1.5
	Y	1793.09		P$_3$•HexN$_2$•C12$_2$•C12OH$_2$•C14OH$_2$	1793.092	1.0
	Y	1713.123		P$_2$•HexN$_2$•C12$_2$•C12OH$_2$•C14OH$_2$	1713.125	1.5
ΔlptA-ΔlpxL1	B	1369.405	PE$_1$•Hex$_1$•Hep$_2$•HexNAc$_1$•Kdo$_2$•Gly$_1$		1369.406	0.4
	B	1312.38	PE$_1$•Hex$_1$•Hep$_2$•HexNAc$_1$•Kdo$_2$		1312.384	3.1
	B-Kdo-CO2	1105.356	PE$_1$•Hex$_1$•Hep$_2$•HexNAc$_1$•Kdo$_2$•Gly$_1$		1105.357	1.3
	B-Kdo-CO2	1048.335	PE$_1$•Hex$_1$•Hep$_2$•HexNAc$_1$•Kdo$_2$		1048.336	0.9
	B-Kdo-CO2	1267.409	PE$_1$•Hex$_2$•Hep$_2$•HexNAc$_1$•Kdo$_2$•Gly$_1$		1267.410	1.0
	B-Kdo-CO2	1210.388	PE$_1$•Hex$_2$•Hep$_2$•HexNAc$_1$•Kdo$_2$		1210.389	0.7
	Y	1610.923		P$_3$•HexN$_2$•C12$_1$•C12OH$_2$•C14OH$_2$	1610.925	1.1
ΔlptA-pagL	B	1369.404	PE$_1$•Hex$_1$•Hep$_2$•HexNAc$_1$•Kdo$_2$•Gly$_1$		1369.406	1.1
	B	1312.382	PE$_1$•Hex$_1$•Hep$_2$•HexNAc$_1$•Kdo$_2$		1312.384	1.6
	B-Kdo-CO2	1105.356	PE$_1$•Hex$_1$•Hep$_2$•HexNAc$_1$•Kdo$_2$•Gly$_1$		1105.357	1.3
	B-Kdo-CO2	1048.335	PE$_1$•Hex$_1$•Hep$_2$•HexNAc$_1$•Kdo$_2$		1048.336	0.9
	B-Kdo-CO2	1267.409	PE$_1$•Hex$_2$•Hep$_2$•HexNAc$_1$•Kdo$_2$•Gly$_1$		1267.410	1.0

Bacteria	Fragment ion type[a)]	Measured mass (*u*)	Proposed LPS composition		Calculated mass (*u*)	Deviation (*ppm*)
			**Oligosaccharide**	**Lipid A**		
	B-Kdo-CO2	1210.387	PE$_1$•Hex$_2$•Hep$_2$•HexNAc$_1$•Kdo$_2$		1210.389	1.5
	Y	1594.928		P$_3$•HexN$_2$•C12$_2$•C12OH$_1$•C14OH$_2$	1594.930	1.2
	Y	1514.961		P$_2$•HexN$_2$•C12$_2$•C12OH$_1$•C14OH$_2$	1514.964	1.7
	Y	1793.092		P$_3$•HexN$_2$•C12$_2$•C12OH$_2$•C14OH$_2$	1793.092	0.1
	Y	1713.125		P$_2$•HexN$_2$•C12$_2$•C12OH$_2$•C14OH$_2$	1713.125	0.3
ΔlptA-LpxE	B	1369.404	PE$_1$•Hex$_1$•Hep$_2$•HexNAc$_1$•Kdo$_2$•Gly$_1$		1369.406	1.1
	B	1312.382	PE$_1$•Hex$_1$•Hep$_2$•HexNAc$_1$•Kdo$_2$		1312.384	1.6
	B	1474.436	PE$_1$•Hex$_2$•Hep$_2$•HexNAc$_1$•Kdo$_2$		1474.437	0.6
	B-Kdo-CO2	1105.355	PE$_1$•Hex$_1$•Hep$_2$•HexNAc$_1$•Kdo$_2$•Gly$_1$		1105.357	2.2
	B-Kdo-CO2	1048.335	PE$_1$•Hex$_1$•Hep$_2$•HexNAc$_1$•Kdo$_2$		1048.336	0.9
	B-Kdo-CO2	1210.387	PE$_1$•Hex$_2$•Hep$_2$•HexNAc$_1$•Kdo$_2$		1210.389	1.5
	Y	1713.124		P$_2$•HexN$_2$•C12$_2$•C12OH$_2$•C14OH$_2$	1713.125	0.9

In-source collision-induced dissociation of LPS produced B- and Y-type fragment ions corresponding to oligosaccharide and lipid A domains due to the rupture of the glycosidic bond between Kdo and lipid A. Fragment ions are assigned according to the nomenclature of Domon and Costello [1]. Mass numbers given refer to monoisotopic masses of the neutral molecules. Abbreviations: Kdo, 3-deoxy-D-*manno*-oct-2-ulosonic acid; Hep, L-*glycero*-D-*manno*-heptose; Hex, hexose; HexNAc, *N*-acetylhexosamine; Gly, glycine; PEA, phosphoethanolamine; P, phosphate; C12OH, 3-hydroxy-dodecanoic acid; C14OH, 3-hydroxy-tetradecanoic acid; C12, dodecanoic acid; C16:1, 9-hexadece

1 Domon, B. & Costello, C. E. A Systematic Nomenclature for Carbohydrate Fragmentations in FAB-MS/MS Spectra of Glycoconjugates. *Glycoconjugate J* **5**, 397-409, doi:10.1007/BF01049915 (1988).
